# A widespread methylotroph acyl-homoserine lactone synthase produces a new quorum sensing signal that regulates swarming in *Methylobacterium fujisawaense*

**DOI:** 10.1128/mbio.01999-23

**Published:** 2023-12-12

**Authors:** Mike Wallace, Dale A. Cummings, Jr., Andrew G. Roberts, Aaron W. Puri

**Affiliations:** 1 Department of Chemistry, University of Utah, Salt Lake City, Utah, USA; 2 Henry Eyring Center for Cell and Genome Science, University of Utah, Salt Lake City, Utah, USA; Instituto Gulbenkian de Ciência, Oeiras, Portugal

**Keywords:** methylotroph, quorum sensing, acyl-homoserine lactone, chemical ecology, swarming motility

## Abstract

Pink-pigmented facultative methylotrophs of the genera *Methylorubrum* and *Methylobacterium* are metabolically versatile bacteria that colonize many diverse environments on earth. Despite their widespread occurrence, the molecular details of how these organisms interact with each other and their environment remain understudied. We analyzed genes encoding *N*-acylhomoserine lactone (acyl-HSL) quorum sensing signal synthases in the published genomes of these bacteria and determined that the product of the largest family of signal synthases had not been characterized. We identified 3*R*-OH-5*Z*-C_12:1_-HSL as a novel signal produced by these synthases using inverse stable isotopic labeling and structural characterization by mass spectrometry, nuclear magnetic resonance spectroscopy, and chemical synthesis. We show that in the methylotroph *Methylobacterium fujisawaense* DSM5686, this signal activates its cognate LuxR-family transcription factor and is produced in a positive feedback loop. We also discover that in this strain, quorum sensing negatively regulates swarming motility by activating the expression of a small protein that binds a predicted transcription factor. These results characterize a previously undescribed, yet widespread quorum sensing system used by pink-pigmented facultative methylotrophs, which helps us understand the chemical ecology of these important bacteria.

**IMPORTANCE:**

Bacteria known as pink-pigmented facultative methylotrophs colonize many diverse environments on earth, play an important role in the carbon cycle, and in some cases promote plant growth. However, little is known about how these organisms interact with each other and their environment. In this work, we identify one of the chemical signals commonly used by these bacteria and discover that this signal controls swarming motility in the pink-pigmented facultative methylotroph *Methylobacterium fujisawaense* DSM5686. This work provides new molecular details about interactions between these important bacteria and will help scientists predict these interactions and the group behaviors they regulate from genomic sequencing information.

## INTRODUCTION

The bacterial genera *Methylobacterium* and *Methylorubrum* primarily consist of pink-pigmented facultative methylotrophs (PPFMs) and are widespread in nature and human-constructed environments (1, 2). These bacteria are often associated with plants, both in the phyllosphere (3) and as endophytes (4), and have been shown to have important roles in plant growth promotion (5, 6). Bacteria from these genera have also been identified as the causative agents of some hospital-acquired infections (7). Despite this recognized importance, the molecular details of how PPFMs interact with each other and their environment are understudied.

Many Gram-negative proteobacteria, including PPFMs, use *N*-acylhomoserine lactones (acyl-HSLs) as quorum sensing (QS) signals (8–11). Acyl-HSL signals are produced by LuxI-family synthases and exhibit variation in their acyl group but maintain a common HSL core originating from methionine (12, 13). Acyl-HSLs are bound by LuxR-family transcription factors that subsequently regulate gene expression to coordinate group behaviors (10), including biofilm formation (14), antibiotic production (15), and swarming motility (16–18). Swarming is a coordinated movement of cells across a surface and relies on three factors: surfactant production and excretion, flagella biosynthesis, and cell-to-cell contact (19). Although swarming can be regulated by QS, the swarming phenotype is not universally associated with high or low cell density, as there are examples of bacteria swarming at both low and high cell densities (16–18). Characterizing PPFM QS systems and the group behaviors they regulate can help us understand the molecular details of PPFM interactions.

We recently reported the use of an inverse stable isotopic labeling (InverSIL) approach to identify methylotroph acyl-HSL QS signals (20). In this approach, we grow a bacterial strain on a ^13^C-labeled version of its carbon source to generate a fully ^13^C-labeled culture. We can then feed this culture ^12^C-precursors and detect their incorporation into natural products by mass spectrometry, thereby eliminating problems with ^13^C-labeled precursor availability. In the case of PPFM acyl-HSLs, bacteria are grown on ^13^C-methanol and then fed ^12^C-methionine, which is incorporated into the HSL portion of these signals.

In this work, we identified a widespread family of acyl-HSL synthases in the publicly available genomes of the genera *Methylobacterium* and *Methylorubrum*. We then used InverSIL to identify and characterize the acyl-HSL product of this synthase family and confirmed this result with multiple acyl-HSL synthase representatives from different strains. When we isolated and determined the structure of this compound, it revealed a signal that, to our knowledge, had not previously been reported. In *Methylobacterium fujisawaense* DSM5686, we then demonstrate that this acyl-HSL signal activates its cognate LuxR-family transcription factor and is produced in a positive feedback loop. In this strain, the signal activates the production of a small protein that regulates swarming behavior by interacting with a predicted transcription factor. These findings provide new information about the chemical ecology of these ubiquitous bacteria.

## RESULTS

### A widespread methylotroph acyl-HSL synthase produces an uncharacterized product

**FIG 1:**
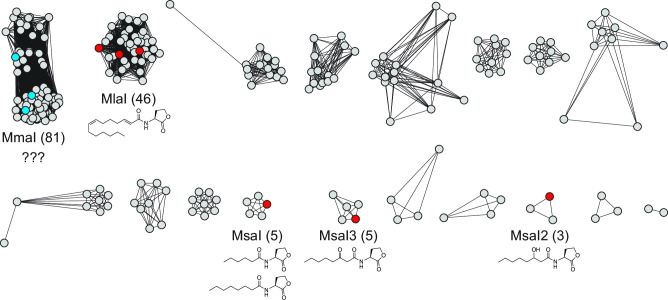
A sequence similarity network of the 271 acyl-HSL synthases in 200 *Methylobacterium* and *Methylorubrum* genomes reveals that the largest synthase family has an unknown product. Red nodes highlight acyl-HSL synthases with previously characterized products. The structure of the major product is shown when reported. Cyan nodes highlight acyl-HSL synthases characterized in this work, here named MmaI. The number of nodes in a cluster is indicated in parentheses. Twenty-one singletons are not shown. Grey nodes: other synthases in each cluster.

To determine the diversity of acyl-HSL QS signals produced by PPFMs, we organized the 271 *Methylobacterium*/*Methylorubrum* LuxI-family acyl-HSL synthases in the Joint Genome Institute’s Integrated Microbial Genomes and Microbiomes (IMG/M) system (21) using sequence similarity networking (22, 23), as has been done previously with acyl-HSL synthases (24). We used an alignment score threshold of 85, which produces isofunctional groups of acyl-HSL synthases (Amy Schaefer and E. Peter Greenberg, University of Washington, personal communication) (Fig. 1). In other words, all members of each synthase cluster are predicted to produce the same acyl-HSL signal. While some clusters contain synthases that have been characterized (MlaI, MsaI1-3) (8, 20), the largest cluster does not have a characterized representative, and therefore a widely conserved signal in this group of organisms cannot be predicted.

**FIG 2:**
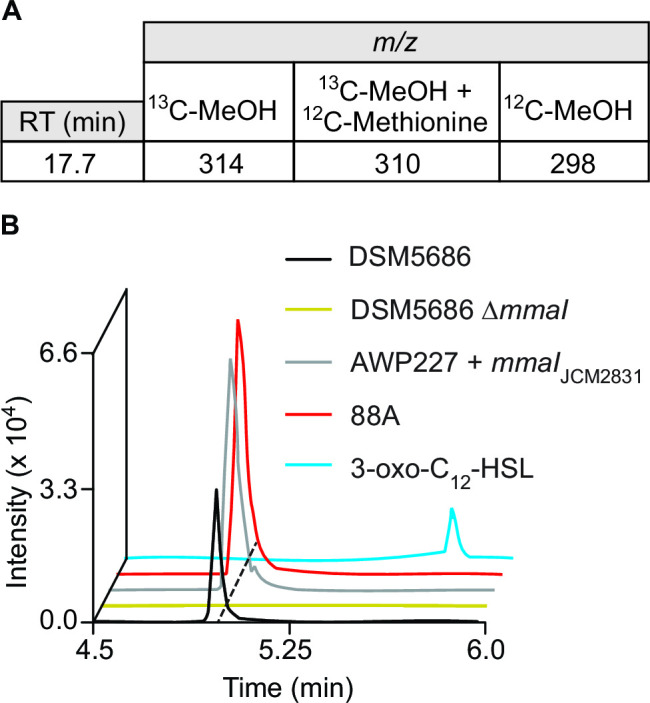
A metabolite that incorporates methionine is produced by multiple PPFM strains that contain the acyl-HSL synthase MmaI. (**A**) InverSIL of strain 88A identifies a feature that incorporates four carbons from methionine. (**B**) Extracted ion chromatogram of supernatant extracts for the listed strains for m/z 298.2013, corresponding to protonated 3*R*-OH-5*Z*-C_12:1_-HSL. Mass tolerance <5 ppm.

PPFM strains often contain multiple annotated *luxI*-family synthase genes in their genomes (9, 25), which can make it difficult to link the production of an acyl-HSL to a specific synthase. *Methylobacterium* sp. strain 88A (26) possesses only one synthase, and it is found in the largest cluster, enabling us to unambiguously link this synthase with its product using InverSIL. When we grew strain 88A on ^13^C-methanol, we identified a feature with a *m/z* of 314 that decreased by four *m/z* units when we included ^12^C-methionine in the media (Fig. 2A). This is consistent with the incorporation of four carbon atoms from methionine into a HSL. In the ^12^C-methanol control, this feature had a *m/z* of 298, indicating this feature likely corresponds to a protonated acyl-HSL containing 16 carbons total, with 12 present in the acyl chain and four in the homoserine lactone ring.

To verify this result, we also examined the products of other representatives of this acyl-HSL synthase cluster using high-resolution LC-MS/MS. When we heterologously expressed the synthase representative from *Methylobacterium radiotolerans* JCM2831 (27) (Mrad2831_5763), which is also present in the largest synthase cluster, we observed a feature with the same *m/z*, retention time, and fragmentation pattern as the feature from strain 88A (Fig. 2B; Table S1). We also observed this feature in a culture of *Methylobacterium fujisawaense* DSM5686 (28) which contains a representative synthase from this cluster (Ga0373204_3345), and this feature was not present in a culture of an in-frame, unmarked deletion mutant of this synthase (Fig. 2B). Together, these results indicate that this feature is the product of the largest cluster of PPFM acyl-HSL synthases (Fig. 1).

The observed high-resolution *m/z* of the feature is consistent with the known signal *N*-(3-oxododecanoyl)-*L*-homoserine lactone (3-oxo-C_12_-HSL) (observed [M + H]^+^ = 298.2013, expected [M + H]^+^ = 298.2013, ∆0.0 ppm). However, a 3-oxo-C_12_-HSL standard exhibited a longer retention time (Fig. 2B), indicating that the target acyl-HSL signal product is likely a constitutional isomer of 3-oxo-C_12_-HSL that, to our knowledge, has not been previously described. We therefore decided to isolate and structurally characterize this compound.

### Characterization of the acyl-HSL product of the largest synthase cluster reveals a new structure

**FIG 3:**
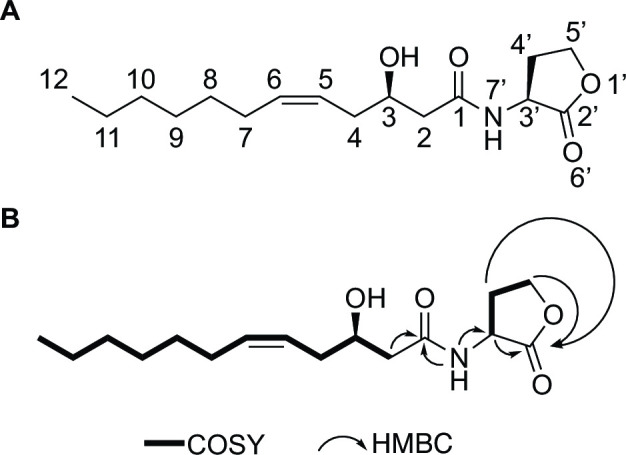
The MmaI acyl-HSL synthase produces a new QS signal. (**A**) Structure of 3*R*-OH-5*Z*-C_12:1_-HSL. (**B**) 2D NMR structural assignments.

We chose *M. fujisawaense* DSM5686 to scale up production of the target acyl-HSL. We grew 30 one-liter cultures and extracted the supernatant with acidified ethyl acetate. The dried extract was then separated by C_18_ solid-phase extraction and purified by HPLC guided by the compound mass. To increase the signal-to-noise ratio for structural elucidation using NMR spectroscopy, we also grew three 1-L cultures of strain DSM5686 using ^13^C-methanol as the sole carbon source. We elucidated the planar structure using ^1^H, ^13^C, and various 2D NMR spectroscopy experiments (Fig. 3; Fig. S1–S6 and Table S2). This analysis revealed a hydroxyl group on carbon 3 and an olefin between carbons 5 and 6 of the aliphatic chain. The ^3^*J*_HH_-coupling of 8.9 Hz enabled us to tentatively assign the olefin in a *cis* configuration.

We next determined the absolute stereochemistry of this compound. Advanced Marfey’s analysis (29) revealed an *L*-HSL (Table S3), which is typical for acyl-HSL QS signals (30). To determine the stereochemistry of the secondary alcohol substituent, we first reduced the olefin in the natural signal to create 3-OH-C_12_-HSL, which we then subjected to methanolysis (31) to produce methyl 3-hydroxydodecanoate (Fig. S7). Using chiral gas chromatography, we compared this methyl ester to commercially available racemic methyl 3-hydroxydodecanoate as well as methyl 3*R*-hydroxydodecanoate, which we synthesized (Fig. S8) (32). This analysis showed the hydroxyl in the *R* configuration (Fig. S9), which has also been noted for other acyl-HSL signals where stereochemistry has been determined (31, 33). Together, this demonstrates that the acyl-HSL product of the largest cluster of PPFM acyl-HSL synthases is *N*-(3*R*-hydroxy-5-*cis*-dodecenoyl)-*L*-homoserine lactone (3*R*-OH-5*Z*-C_12:1_-HSL) (Fig. 3). We therefore name the cluster of synthases that produces this acyl-HSL signal MmaI, for *Methylobacterium*/*Methylorubrum* medium-chain acyl-HSL.

### 3*R*-OH-5*Z*-C_12:1_-HSL activates the transcription factor MmaR

**FIG 4:**
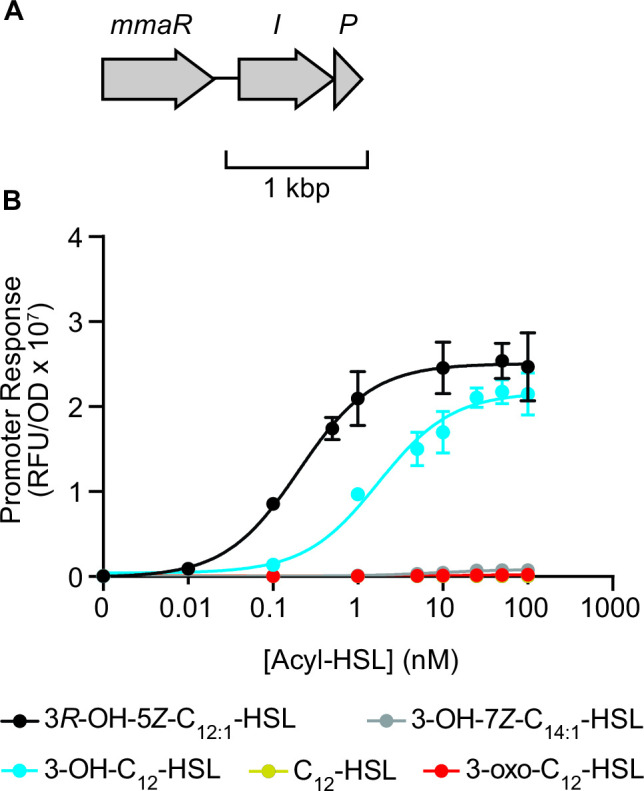
The transcription factor MmaR is specific for its cognate signal, 3*R*-OH-5*Z*-C_12:1_-HSL. (**A**) Mma QS gene neighborhood in strain DSM5686. (**B**) Activation of the *mmaI*_DSM5686_ promoter fused to the reporter gene *mScarlet* by acyl-HSL signals in the reporter strain AWP370 expressing the *mmaR*_DSM5686_ transcription factor. RFU: relative fluorescence units. OD: optical density at 600 nm. Data show the mean and standard deviation of five cultures and are representative of two independent experiments.

To determine if the signal is bioactive, we constructed an acyl-HSL QS reporter strain (34). The *mmaI* gene is co-located in the genome of strain DSM5686 with a gene encoding a LuxR-family transcription factor (Ga0373204_3344), which we term *mmaR* (Fig. 4A). To construct the reporter strain AWP370, we first inserted *mmaR* into the genome of the heterologous expression strain *Methylorubrum extorquens* AWP227, where the endogenous QS genes *mlaR* and *mlaI* have been deleted (20), downstream of the native *mlaR* promoter. We then introduced a plasmid containing the red fluorescent reporter gene *mScarlet*, driven by the *mmaI* upstream region from strain DSM5686. LuxI-family acyl-HSL synthases are often positively autoregulated by their cognate LuxR-family transcription factors (11, 34), and therefore we hypothesized mScarlet would be expressed when the signal is added to AWP370.

We observed an increase in red fluorescence with an EC_50_ of 0.2 nM when we added various concentrations of the signal to the reporter strain AWP370 (Fig. 4B). Early stationary phase cultures of the wild-type (WT) DSM5686 contain approximately 700 nM of signal, or over 1,000-fold more than is required to activate this heterologous reporter. We also determined that this signal is produced when we grow DSM5686 on methanol but not the multicarbon substrate succinate (Fig. S10), as has been previously observed for acyl-HSL QS signals produced by MlaI in *Methylorubrum extorquens* AM1 (8).

When we tested four structurally related, commercially available acyl-HSLs (3-oxo-C_12_-HSL, 3-OH-C_12_-HSL, C_12_-HSL, and 3-OH-7Z-C_14:1_-HSL) with the reporter strain AWP370, only 3-OH-C_12_-HSL activated MmaR at a concentration similar to the native signal (EC_50_ 1.7 nM) (Fig. 4B). Together, these results demonstrate that *3R*-OH-5*Z*-C_12:1_-HSL can activate the transcription factor MmaR_DSM5686_ and that this signal is produced in a positive feedback loop.

### Mma QS suppresses swarming in *Methylobacterium fujisawaense* DSM5686 by activating the expression of a small protein, MmaP

**FIG 5:**
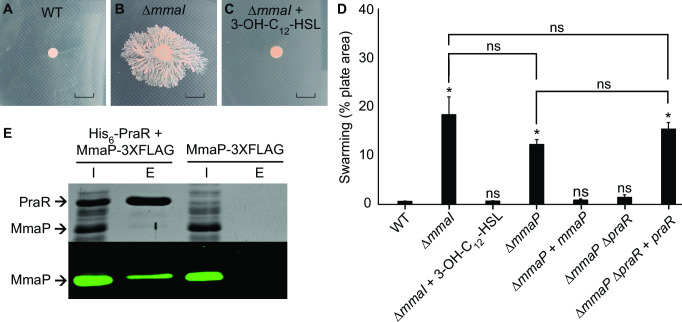
The Mma QS system regulates swarming through the expression of the small protein, MmaP, which in turn binds to the predicted transcription factor, PraR. (**A–C**) Mma QS negatively regulates swarming in strain DSM5686. The scale bar represents 10 mm. (**D**) Quantification of swarming motility in DSM5686 strains. Data show the mean and standard deviation of three plates and are representative of two independent experiments. Means were compared to WT using a one-way ANOVA with Tukey’s *post hoc* test. *, *P* < 0.001; ns, not significant. (E, top) Coomassie staining of IMAC input and elution fractions with and without His_6_-PraR present. (E, bottom) Anti-FLAG western blot of IMAC input and elution fractions. The input was diluted 1/10 for the Coomassie staining and 1/200 for the western blot relative to the other lanes. “I” refers to input, and “E” refers to elution.

While assaying strains DSM5686 and DSM5686 ∆*mmaI* for phenotypes commonly regulated by QS, we found that strain DSM5686 Δ*mmaI* swarms on soft agar but WT DSM5686 does not (Fig. 5A and B). To verify that swarming is a result of the deletion of *mmaI*, we complemented strain DSM5686 Δ*mmaI* by adding 3-OH-C_12_-HSL to the agar and found that the mutant strain no longer swarmed (Fig. 5C). These results indicate that the Mma QS system negatively regulates swarming in DSM5686.

Immediately downstream of *mmaI* in the genome of strain DSM5686 is a small open reading frame encoding a 59 amino acid protein (Fig. 4A). We also found a similar downstream open reading frame in many other PPFM genomes that contain *mmaI* (Fig. S11) (35), leading us to predict that this gene is regulated by QS. We used RT-PCR to confirm that *mmaI* and this open reading frame, which we refer to as *mmaP*, are co-transcribed (Fig. S12), indicating that transcription of *mmaP* is activated by QS.

To determine if MmaP affects swarming in *M. fujisawaense* DSM5686, we created the deletion mutant DSM5686 Δ*mmaP* and found that it swarmed (Fig. 5D), even though this strain still produces 3*R*-OH-5*Z*-C_12:1_-HSL (Fig. S13). To verify that swarming was a result of *mmaP* deletion, we complemented DSM5686 Δ*mmaP* with a plasmid-borne copy of *mmaP* under the QS-regulated *mmaI* promoter and found that the mutant strain no longer swarmed (Fig. 5D). These results indicate that the Mma QS system suppresses swarming through the expression of MmaP in *M. fujisawaense* DSM5686.

### The predicted transcription factor PraR positively regulates swarming in *M. fujisawaense* DSM5686 and binds MmaP

Given its small size and lack of annotated catalytic or DNA-binding domains, we hypothesized that MmaP represses swarming by binding another protein in strain DSM5686. The Downie Lab previously reported that a QS system in *Rhizobium leguminosarum* bv. viciae also regulates swarming via a small protein, CinS, that is cotranscribed with the acyl-HSL synthase CinI (16, 36, 37). CinS activates swarming by binding a transcriptional repressor, PraR. We hypothesized that a similar system could be in place in strain DSM5686, although in this system the WT strain does not swarm. A BLAST search identified a potential PraR-ortholog in the genome of strain DSM5686 (Ga0373204_957). While this gene product only shares ~35% amino acid identity to PraR in *R. leguminosarum*, its gene has the same two genes downstream in the DSM5686 genome: a predicted *S*-adenosyl methionine synthetase gene (Ga0373204_956) and a predicted tRNA-methyltransferase gene (Ga0373204_955). Additionally, all PPFM strains that contain the Mma QS system also contain this *praR*-ortholog, with the same two genes downstream (Fig. S14). The similarity of these gene neighborhoods, in conjunction with the co-occurrence of this gene with the Mma QS system, made the product of Ga0373204_957, now PraR, a likely PraR-ortholog.

To investigate the role of PraR in the swarming of strain DSM5686, we deleted *praR* in the WT and Δ*mmaP* strains to create strains DSM5686 Δ*praR* and DSM5686 Δ*mmaP* Δ*praR*, respectively, and assayed these strains for swarming. We found that deletion of *praR* had no effect on swarming in the WT strain, but that deletion of *praR* from Δ*mmaP* caused it to cease swarming (Fig. 5D). We could complement this phenotype with a plasmid-borne copy of *praR* under its native promoter (Fig. 5D). These results indicate that PraR is downstream in the same swarming pathway as QS-regulated MmaP and that it positively regulates swarming.

In *R. leguminosarum*, CinS binds PraR. To determine if MmaP and PraR directly interact in DSM5686, we performed a pull-down assay with His_6_-tagged PraR and 3XFLAG-tagged MmaP. We first confirmed that a C-terminally 3XFLAG-tagged MmaP still represses swarming in DSM5686 (Fig. S15). We then co-expressed MmaP-3XFLAG with His_6_-PraR in *Escherichia coli* and purified His_6_-PraR using immobilized metal affinity chromatography (IMAC) (Fig. 5E). Western blotting against the FLAG epitope revealed that MmaP-3XFLAG copurified with His_6_-PraR (Fig. 5E). No MmaP-3XFLAG was detected in a control experiment where His_6_-PraR was not present. These results lead us to conclude that PraR and MmaP bind.

## DISCUSSION

PPFMs are ubiquitous, metabolically versatile bacteria with reported interactions with plants, bacteria, and humans. Here we identified and characterized the Mma QS system, which is widely conserved among PPFMs of the genera *Methylobacterium* and *Methylorubrum*. Characterized straight-chain acyl-HSLs vary in chain length and may also contain a hydroxyl or carbonyl at the third carbon of the chain and/or one or more double bonds, which results in signal structures with specificity for their cognate QS systems (34). We determined that the Mma QS system produces and responds to the signal 3*R*-OH-5*Z*-C_12:1_-HSL, which contains a combination of these structural features that, to our knowledge, has not been previously described. The only other reported acyl-HSL containing both a double bond and hydroxyl is 3-OH-7*Z*-C_14:1_-HSL, produced by *R. leguminosarum* (33), but MmaR does not respond appreciably to this signal (Fig. 4B).

**FIG 6:**
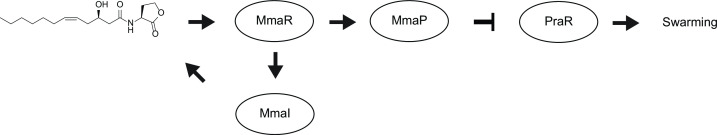
Proposed Mma QS signaling pathway that negatively regulates swarming motility in *M. fujisawaense* DSM5686. The acyl-HSL synthase MmaI produces the signal 3*R*-OH-5*Z*-C_12:1_-HSL, which in turn binds and activates the transcription factor MmaR. MmaR then upregulates the production of the small protein MmaP, which binds and inhibits PraR, a predicted transcription factor that upregulates swarming in strain DSM5686 when not inhibited by MmaP.

We examined the role of the Mma QS system in *M. fujisawaense* DSM5686 and discovered that it negatively regulates swarming in this strain. Swarming motility has been reported for other PPFM strains isolated from drinking water (38) and the phyllosphere (39); however, to our knowledge, the connection between swarming and QS in PPFMs has not been previously investigated. Our results are consistent with Mma QS activating production of the small protein MmaP, which in turn binds the predicted transcription factor PraR. Because the presence of PraR is positively correlated with swarming in the absence of MmaP (Fig. 5D), PraR could activate swarming and be inhibited by MmaP (Fig. 6). This mechanism is similar to the Cin QS system that has been characterized in *R. leguminosarum* bv. viciae; however, the Cin QS system positively regulates swarming.

We observed that in DSM5686, methanol leads to the activation of the Mma QS system (Fig. S10), which means that this environmental cue halts swarming in this strain. This is consistent with these bacteria swarming until they locate a source of the one carbon substrate, methanol, at which point QS would be activated and swarming would stop. This could serve a similar role to more well-studied bacterial chemotaxis systems. Conversely, inhibition of the Mma QS system by other QS signals could be a cue for PPFMs to move away from other bacteria. While the type of environment *M. fujisawaense* DSM5686 was isolated from is unclear, swarming is known to be important for plant colonization (18, 40). Because many PPFMs are plant-associated, the connection between Mma QS in PPFMs, swarming, and plant colonization warrants future attention.

Our analysis of the QS systems in *Methylobacterium* and *Methylorubrum* also shows that there are many other families of acyl-HSL synthases in PPFMs left to be characterized (Fig. 1). Some of these acyl-HSL synthase families may also produce signals with new structures. Our strategy of inserting *luxR*-family transcription factor genes into AWP227 and then testing various acyl-HSL signals and plasmid-contained receptor binding sites is also generalizable and can be used to characterize more LuxR-family transcription factors from these PPFM QS systems in the future. This characterization would help identify potential signal overlap between PPFMs and other bacteria in different environments. The discovery of new orthogonal acyl-HSL signaling systems can also help synthetic biologists use exogenous signals to encode orthogonal regulatory circuits in organisms of interest (41).

## MATERIALS AND METHODS

### Routine bacterial culturing

Strains used in this study are listed in Table S4. *E. coli* strains were grown in lysogeny broth (LB) at 37°C. *M. extorquens* AWP227 derivatives, *Methylobacterium* sp. strain 88A, and *M. fujisawaense* DSM5686 were grown at 30°C in modified ammonium mineral salts (AMS) medium (42), with the addition of 0.01% (wt/vol) yeast extract for 88A and DSM5686. Modified AMS contains 0.2 g L^−1^ MgSO_4_·7H_2_O, 0.2 g L^−1^ CaCl_2_·6H_2_O, 0.5 g L^−1^ NH_4_Cl, 30 µM LaCl_3_, and 1X trace elements. 500× trace elements contains 1.0 g L^−1^ Na_2_-EDTA, 2.0 g L^−1^ FeSO_4_·7H_2_O, 0.8 g L^−1^ ZnSO_4_·7H_2_O, 0.03 g L^−1^ MnCl_2_·4H_2_O, 0.03 g L^−1^ H_3_BO_3_, 0.2 g L^−1^ CoCl_2_·6H_2_O, 0.6 g L^−1^ CuCl_2_·2H_2_O, 0.02 g L^−1^ NiCl_2_·6H_2_O, and 0.05 g L^−1^ Na_2_MoO·2H_2_O. A final concentration of 4 mM phosphate buffer pH 6.8 and 50 mM ^12^C- or ^13^C-methanol, or 15 mM succinate, were added prior to use, and cultures were shaken at 200 rpm.

### Acyl-HSL synthase sequence similarity networking

On January 27, 2022, all genomes of the genera *Methylobacterium* and *Methylorubrum* were downloaded from the JGI IMG/M system (21), returning 200 genomes. These genomes were then searched for genes containing pfam00765, returning 271 genes. Amino acid sequences were exported in FASTA format and analyzed using the Enzyme Function Initiative SSN workflow (23) using an alignment score threshold of 85. The network was visualized without collapsing nodes containing 100% amino acid identity with Cytoscape 3.9.1 (43) using the Prefuse Force Directed OpenCL Layout by alignment score.

### Genetic manipulation

All gene locus tags in this manuscript refer to the JGI IMG/M system (21). Genetic manipulation of strains *M. extorquens* AWP227 and *M. fujisawaense* DSM5686 was performed at 30°C as previously described (20, 44). Sequence-verified plasmids were conjugated into these strains using the *E. coli* donor S17-1 (45). In addition, 500 µL of exponentially growing cultures (OD 0.4–0.6) of the donor and recipient strains were pelleted at 16,100 rcf for 1 minute and resuspended in 500 µL sterile ultrapure H_2_O. These strains were then pelleted again, and the two pellets were combined in a total volume of 50 µL sterile ultrapure H_2_O. Next, the entire mixture was spotted onto a modified AMS agar plate containing 50 mM methanol and 10% (vol/vol) nutrient broth and incubated for 2 days at 30°C. Successful transconjugants were selected on modified AMS plates containing kanamycin (50 µg mL^−1^). To construct the unmarked insertion mutants, kanamycin-resistant integrants (single crossovers) were restreaked and then plated on a modified AMS plate containing 50 mM methanol and 1% (m/v) sucrose for counterselection. The resulting colonies were screened for double crossovers by kanamycin sensitivity and colony PCR before the final mutant was verified by Sanger sequencing.

### Large-scale production and purification of 3*R*-OH-5*Z*-C_12:1_-HSL

An exponentially growing culture of *M. fujisawaense* DSM5686 was used to inoculate one L modified AMS + 50 mM MeOH to a starting optical density of 0.02. A total of 6 1 L cultures were grown at a time. These cultures were incubated at 30°C and shaken at 200 rpm to stationary phase (approximately 72 hours). The culture was centrifuged at 4,700 rpm for 10 min followed by extraction of the clarified supernatant with an equal volume of ethyl acetate acidified with 0.01% (vol/vol) acetic acid. The organic phase was then dried by rotary evaporation. Dried crude supernatant extracts were resuspended in 1 mL of 1:3 water:acetonitrile (ACN) and applied onto a preequilibrated Discovery C_18_ solid-phase extraction (SPE) column (3 mL, 500 mg) then washed and eluted sequentially with 6 mL of 25%, 50%, 75%, and 100% ACN in water. The acyl-HSL was found in the 75% ACN fraction by LC-MS. This fraction was dried and resuspended in 500 µL of 25% ACN in water and separated using an Agilent 1260 Infinity liquid chromatography system. A Waters SunFire C18 column (3.5 µm particle size, 46 mm × 100 mm) was used for reverse phase separation with a flow rate of 1 mL min^−1^. Solvent A: Water + 0.1% trifluoroacetic acid; Solvent B: ACN + 0.1% trifluoroacetic acid. Gradient: 0–1 min, 10–35% B. 1–21 min, 35–55% B. 21–22 min, 55–100% B. 22–30 min, 100% B. 30–31 min, 100–10% B. 31–36 min, 10% B.

### 3*R*-OH-5*Z*-C_12:1_-HSL

HRESIMS: [M + H]^+^ = 298.2013 (calculated for C_16_H_28_NO_4_^+^: 298.2013; 0.0 ppm). MS2: see Table S1. ^1^H NMR (800 MHz, CDCl_3_) and ^13^C NMR (200 MHz, CDCl_3_): see Table S2.

For additional methods, please see supporting information.

## SUPPLEMENTAL MATERIAL

Supplemental material mbio.01999-23-s0001.pdf Methods, Fig. S1-S15, and Tables S1-S6.

ASM does not own the copyrights to Supplemental Material that may be linked to, or accessed through, an article. The authors have granted ASM a non-exclusive, world-wide license to publish the Supplemental Material files. Please contact the corresponding author directly for reuse.
