# Supplementary material for: A widespread methylotroph acyl-homoserine lactone synthase produces a new quorum sensing signal that regulates swarming in Methylobacterium fujisawaense
Source: mBio. 2023 Dec 12;15(1):e01999-23. doi: 10.1128/mbio.01999-23 (PMC10790750; doi:10.1128/mbio.01999-23)
Supplement: Supplemental material — Methods, Fig. S1-S15, and Tables S1-S6. [file mbio.01999-23-s0001.pdf]

SUPPLEMENTARY INFORMATION

A widespread methylotroph acyl-homoserine lactone synthase produces a new quorum sensing signal that regulates swarming in *Methylobacterium fujisawaense*

Mike Wallace[1,2], Dale A. Cummings Jr.[1,2#], Andrew G. Roberts[1], and Aaron W. Puri[1,2*]

[1]Department of Chemistry, University of Utah, Salt Lake City, Utah, USA
[2]Henry Eyring Center for Cell and Genome Science, University of Utah, Salt Lake City, Utah, USA

#Present address:
Dale A. Cummings Jr., Department of Chemistry and Biochemistry, Northern Arizona University, Flagstaff, Arizona, USA

*Corresponding author:
Aaron W. Puri
315 S 1400 E Rm 2020
Salt Lake City, UT 84112
USA
(801) 213-1408
a.puri@utah.edu

TABLE OF CONTENTS

SUPPLEMENTARY METHODS 3

Key reagents. 3
Inverse stable isotopic labeling (InverSIL) experiments. 3
LC-MS for acyl-HSL signal detection. 3
Inverse stable isotopic labeling (InverSIL) analysis. 3
Plasmid construction. 3
High-resolution tandem mass spectrometry. 4
Marfey's analysis of 3*R*-OH-5*Z*-$C_{12:1}$-HSL. 4
Catalyst preparation for synthesis of 3*R*-hydroxydodecanoic acid methyl ester 4
Reduction of methyl 3-oxododecanoate to 3*R*-hydroxydodecanoic acid methyl ester (2). 4
Derivatization of natural 3*R*-OH-5*Z*-$C_{12:1}$-HSL. 4
Chiral gas chromatography analysis. 5
$MmaR_{DSM5686}$ reporter assay. 5
Swarming assay. 5
Expression of His_6-PraR and MmaP-3XFLAG. 5
His_6-PraR pull-down. 5
Western blot detection of purified MmaP-3XFLAG. 6

SUPPLEMENTARY FIGURES 7

Figure S1. 7
Figure S2. 8
Figure S3. 9
Figure S4. 10
Figure S5. 11
Figure S6. 12
Figure S7. 13
Figure S8. 13
Figure S9. 14
Figure S10. 15
Figure S11 16
Figure S12. 17
Figure S13. 17
Figure S14. 18
Figure S15. 19

SUPPLEMENTARY TABLES 20

Table S1 20
Table S2 21
Table S3 21
Table S4 22
Table S5 24
Table S6 25

SUPPLEMENTARY REFERENCES 28

SUPPLEMENTARY METHODS

Key reagents. ^{13}C-labeled methanol was purchased from Cambridge Isotope Laboratories. 3-OH-C_{12}-HSL was purchased from Millipore Sigma. All other acyl-HSLs were purchased from Cayman Chemical.

Inverse stable isotopic labeling (InverSIL) experiments. Inverse labeling and subsequent analysis was performed as previously described (1). Exponentially growing bacterial cultures were pelleted at 16,100 rcf for one minute and resuspended in growth medium with no carbon source. Subsequently, three separate six milliliter cultures were inoculated with the resuspended strain at a starting OD of 0.02. The ^{12}C-carbon source was added to one culture, the ^{13}C-carbon source to the second, and the ^{13}C-carbon source plus 500 nM ^{12}C-methionine to the last culture. The carbon sources used were 50 mM methanol, 50 mM methylamine, or 50% (v/v) methane. Cultures were grown until reaching stationary phase (OD of approximately 0.8) and then were centrifuged at 4,800 rcf for ten minutes. The resulting supernatant was extracted twice with an equal volume of ethyl acetate containing 0.01% (v/v) acetic acid, and the combined organic extract was evaporated to dryness using a nitrogen stream and stored at -20 ºC until analysis by LC-MS.

LC-MS for acyl-HSL signal detection. Dried culture supernatant extracts were resuspended in 200 microliters of 1:1 water:acetonitrile, and subsequently 65 microliters were injected onto an Agilent 1260 Infinity liquid chromatography system connected to an Agilent 6120 single quadrupole mass spectrometer operating with positive polarity and a mass range of 150-1500 *m/z*. A Waters Xselect HSS T3 column (2.5 µm particle size, 2.1 mm x 50 mm) held at 30 ºC was used for reverse phase separation with a flow rate of 0.4 mL min^{-1}. Solvent A: Water + 0.1 % formic acid, Solvent B: Acetonitrile + 0.1% formic acid. Gradient: 0-2 min, 0% B. 2-32 min, 0-100% B. 32-35 min, 100% B. 35-36 min, 100-0% B. 36-38 min, 0% B.

Inverse stable isotopic labeling (InverSIL) analysis. Inverse labeling analysis was performed as previously described (1). Raw data files in netCDF format were exported using Agilent OpenLab CDS (rev C.01.07). Features were detected using MZmine version 2.53 (2) using the following workflow: 1. Mass detection (centroid, noise level 1.0E3). 2. ADAP chromatogram builder (minimum group size 5 scans, group intensity threshold 1.0E3, min highest intensity 5.0E3), *m/z* tolerance 0.3). 3. Chromatogram deconvolution (local minimum search, chromatogram threshold 30%, search minimum 0.1 min, minimum relative height 10%, minimum absolute height 6.0E3, minimum ratio of peak top/edge 2, peak duration 0-2 min). 4. Adduct search (RT tolerance 0.1 min, adducts $[M+Na]^+$ and $[M+NH_4]^+$ selected, *m/z* tolerance 0.2, max relative peak height 200%). 5. Feature list rows filter (remove identified adducts). Subsequently, isotopes were removed from ^{12}C samples using the Isotopic peaks grouper (*m/z* tolerance 0.2, retention time tolerance 0.1 min, monotonic shape required, maximum charge 3, representative isotope most intense), and the three feature lists were aligned in the order ^{13}C-carbon source + ^{12}C-methionine, ^{12}C-carbon source, ^{13}C-carbon source using the Join aligner (*m/z* tolerance 0.3, weight of *m/z* 50, retention time tolerance 0.1 min, weight for retention time 50). The alignment was exported in .csv format with the row retention time as a common element and peak *m/z* as the data file element. Features containing the desired four *m/z* unit difference in the ^{13}C-carbon source and ^{13}C-carbon source + ^{12}C-methionine samples were then detected using a custom Python script (available at https://github.com/purilab/inverse).

Plasmid construction. Plasmids used in this study are listed in **Table S5**. Primers used in this study are listed in **Table S6**. All plasmids were constructed using Gibson Assembly (4) and selection was performed with kanamycin (50 µg mL^{-1}).

High-resolution tandem mass spectrometry. Mass spectrometry data were collected using a Waters Acquity I-class ultra-high pressure liquid chromatography (UPLC) instrument coupled to a Waters Xevo G2-S quadrupole time-of-flight mass spectrometer. An Acquity UPLC BEH C18 column (2.1 x 50 mm) was used for separation and resolving samples. Solvent A: Water + 0.1 % (v/v) formic acid, Solvent B: Acetonitrile + 0.1% (v/v) formic acid. The sample was eluted from the column using a ten minute linear solvent gradient: 0-0.1 min, 1% B; 0.1 - 10 min, 1-100% B. The solvent flow rate was 0.45 mL min^{-1}. Mass spectra were collected in positive ion mode, with following parameters: 3 kV capillary voltage; 25 V sampling cone voltage; 150 °C source temperature; 500 °C desolvation temperature; nitrogen desolvation at 800 L/hr. The fragmentation spectra were collected using the same parameters with a 10-25 eV collision energy ramp. The lockspray solution was 200 pg/µL leucine enkephalin. The lockspray flow rate was 6 µL/min. Sodium formate was used to calibrate the mass spectrometer.

Marfey's analysis of 3*R*-OH-5*Z*-$C_{12:1}$-HSL. 3*R*-OH-5*Z*-$C_{12:1}$-HSL (0.1mg) was suspended in 250 µL of 6M HCl in water overnight at 110 °C to cleave the amide bond. This reaction was then lyophilized and the resulting solid was resuspended in 250 µL of saturated sodium bicarbonate. 16 µL of 1% (m/v) of Marfey's reagent (Sigma-Aldrich, 71478) was added and the mixture was allowed to react for 1 hour at 40 °C. The reaction was then quenched with 20 µL of 2M HCl in water and analyzed by LC-MS, where an *m/z* of 372 was found, corresponding to the $[M+H]^+$ of the hydrolyzed HSL with a Marfey's adduct. Retention times were compared to Marfey's derivatized *L*- and *R*-HSL standards.

Catalyst preparation for synthesis of 3*R*-hydroxydodecanoic acid methyl ester. The catalyst, (R)-$[RuCl_2(BINAP)]_2 \cdot NEt_3$, was prepared by a procedure adopted from Taber and Silverberg (5). All steps were performed under inert atmosphere. Briefly, 40 mg Dichloro(1,5-cyclooctadiene) ruthenium(II) polymer (Sigma-Aldrich, 337331) and 100 mg (R)-BINAP (Sigma-Aldrich 295187) were dissolved in toluene (5 mL) and triethylamine (0.3 mL) was added. The mixture was refluxed (oil bath at 140 °C) for 4 hours, after which the solvent was removed under vacuum. The resulting red-orange powder was dissolved in 1 mL THF and immediately used in the next reaction.

Reduction of methyl 3-oxododecanoate to 3*R*-hydroxydodecanoic acid methyl ester (2). Methyl 3-oxododecanoate **(1)** (100mg) (TRC, M324720), methanol (0.5 mL) and the above catalyst (0.1 mL) were added to a 1-dram vial. The solution was sparged with N_2, followed by H_2 for 10 minutes each. The reduction was carried out under 1 atm H_2 at 60 °C overnight. Subsequently, the product was purified by silica flash chromatography to give 15 mg of a colorless oil (15% yield). Enantiomeric excess was determined by chiral GC to be 95.7%. LCMS: $[M+H]^+$ = 231. ^{1}H NMR (300 MHz, $CDCl_3$) δ 7.28, 4.06, 4.05, 4.05, 4.04, 4.02, 4.01, 4.00, 3.74, 2.87, 2.58, 2.57, 2.52, 2.51, 2.47, 2.44, 2.42, 2.39, 1.57, 1.54, 1.51, 1.50, 1.47, 1.45, 1.43, 1.33, 1.30, 1.28, 1.27, 0.92, 0.91, 0.90, 0.88. ^{13}C NMR (75 MHz, $CDCl_3$) δ 173.6, 77.4, 77.0, 76.6, 68.0, 51.8, 41.1, 36.5, 31.9, 29.57, 29.55, 29.5, 29.3, 25.5, 22.7, 14.1.

Derivatization of natural 3*R*-OH-5*Z*-$C_{12:1}$-HSL. First, the acyl chain of 3*R*-OH-5*Z*-$C_{12:1}$-HSL **(3)** was reduced with Pd/C to yield 3-OH-C_{12}-HSL **(4)**. 3*R*-OH-5*Z*-$C_{12:1}$-HSL (**3**; 0.2 mg) was dissolved in absolute ethanol (0.1 mL) and sparged with N_2 for 10 minutes. Simultaneously, 5% (m/v) of palladium on carbon (Aldrich, 205699) in absolute ethanol (0.1 mL) was sparged with N_2, followed by sparging with H_2. The solutions were combined and left to react at room temperature for 4 hours, after which time the solvent was removed under a stream of N_2. Using a protocol adapted from Thiel *et al*. (6), the resulting **4** was filtered through cotton, then underwent methanolysis, 2% (v/v) H_2SO_4 in absolute methanol, at 60 °C overnight to yield methyl 3-hydroxydodecanoate **(5)**.

Excess acid was neutralized with sodium bicarbonate, then the solution was filtered through cotton in preparation for GC analysis.

Chiral gas chromatography analysis. To determine the absolute stereochemistry of **5**, chiral GC analysis was performed. Retention times of **2**, racemic 3-hydroxydodecanoic acid methyl ester (TRC, H939600), and **5** were compared. Separation of the *R*/*S* methyl ester enantiomers was performed on an Agilent 6890 GC fitted with an Agilent HP-Chiral column (30m, 0.32mm i.d., Agilent) and a flame ionization detector. 1 µL of each sample was injected in split-injection mode (10:1). The instrument was run at 115 °C isocratic for 90 minutes, followed by an increase to 220 °C (5 °C/min). H_2 carrier gas (4.0 mL/min) was used.

MmaR$_{\mathbf{DSM5686}}$ reporter assay. An overnight culture of AWP370 reporter strain (AWP348+pAWP492) was subcultured to an optical density of 0.05 in fresh AMS containing kanamycin (50 ug/mL), 0.01% (m/v) yeast extract, and 50mM MeOH in a 50 mL sterile conical tube. 0.5 mL of this culture was added to each of the wells of a 96-well deep well plate containing 5 µL of the appropriate acyl-HSL dissolved in acidified ethyl acetate. The plate was then incubated at 30 °C for 24 hours with shaking (200 rpm). 100 µL of each well was transferred to a black with clear flat bottom 96-well plate (Corning 3631) and red fluorescence was quantified at 570 nm excitation and 605 nm emission using a SpectraMax i3x plate reader. Absorbance at 600 nm was also quantified for normalization. Each condition was performed in triplicate. Results were analyzed using Graphpad Prism version 8.0.2.

Swarming assay. 6 ml cultures of DSM5686-derived strains were inoculated from plates and grown to stationary phase plus an additional two days in R2A media supplemented with 50mM MeOH. For strains that were grown on kanamycin, 50 µg mL^{-1} was used. Cultures were normalized by OD and 5 µL of the culture was spotted onto the center of a soft agar R2A plate supplemented with 50 mM methanol, and kanamycin when necessary. Soft agar R2A plates had an agar concentration of 0.4% (m/v) and a volume of 25mL. 10µM 3-OH-C_{12}-HSL was used for complementing the Δ*mmaI* strain. After pouring, plates were left to air dry without lids in a biosafety cabinet for ~20 minutes before spotting. Plates were incubated, inverted, at 30 ºC for 4-6 days before photographing. Swarming area was quantified in ImageJ (https://imagej.net/ij/) by tracing the perimeter of the bacterial swarm and measuring the number of pixels contained within that area. This area was then divided by the total area of the plate to give % plate area.

Expression of His$_6$-PraR and MmaP-3XFLAG. 10mL terrific broth (TB) overnight cultures of BL21 *E. coli* expressing MmaP-3XFLAG with or without His$_6$-PraR were used to inoculate 1L of TB. These cultures were grown at 30ºC to an OD of ~0.5, then induced with a final concentration of 0.5mM IPTG. Induced cultures were grown overnight at 17 ºC. The following morning, two 450 mL portions of the culture were pelleted and the pellets were frozen overnight at -80ºC. The following day, the 450 mL culture pellets were resuspended in 15 mL of lysis buffer (50 mM Tris, 20 mM imidazole, 1 mM PMSF, pH 8.0). 1 mg/mL lysozyme was added and the mixture was left on ice for 30 minutes before sonication (2s on, 2s off for 4 min at 70% amplitude). Cell lysate was clarified for 60 minutes at 21,000 rcf. Protein concentrations were determined by absorbance at 280nm and normalized to the lowest concentration by addition of lysis buffer.

His$_6$-PraR pull-down. 8 mL of clarified lysate was combined with 1 mL of IMAC resin that had been pre-equilibrated in wash buffer (50mM phosphate buffer, 250mM NaCl, 25 mM imidazole, pH 8.0). This mixture was left to equilibrate in at 4 ºC for one hour. The resin/clarified lysate mixture was loaded onto a gravity column and the flow through was discarded. The resin was washed twice with 10 mL wash buffer. The resin was then washed the following number of times with 1mL of wash buffer with increasing concentrations of imidazole: 2 x 100 mM, 2 x 200 mM, 3

x 300 mM. His_6-PraR was then eluted with 8 x 0.5 mL of elution buffer (50mM phosphate buffer, 250mM NaCl, 500 mM imidazole, pH 8.0) and all fractions were analyzed by SDS-PAGE.

Western blot detection of purified MmaP-3XFLAG. Protein samples to be analyzed were denatured in an equal volume of 2X Laemmli SDS sample buffer at 97ºC for six minutes and then separated on a 15% polyacrylamide gel. The gel was then transferred to a 0.2 µm PVDF membrane. The membrane was rinsed twice with PBST and then blocked in a PBST + 5% (m/v) dry milk for 30 minutes at room temperature. The membrane was rinsed twice with PBST, then incubated with the primary antibody overnight at 4ºC (Sigma-Aldrich, F3165 1:3000 in PBST). The following morning, the membrane was washed 3X with PBST and incubated with the secondary antibody (LI-COR, 926-32210, 1:10,000 in PBST) for two hours at room temperature. The membrane was then washed and imaged on the LiCor Odyssey CLx imager at the highest resolution.

SUPPLEMENTARY FIGURES

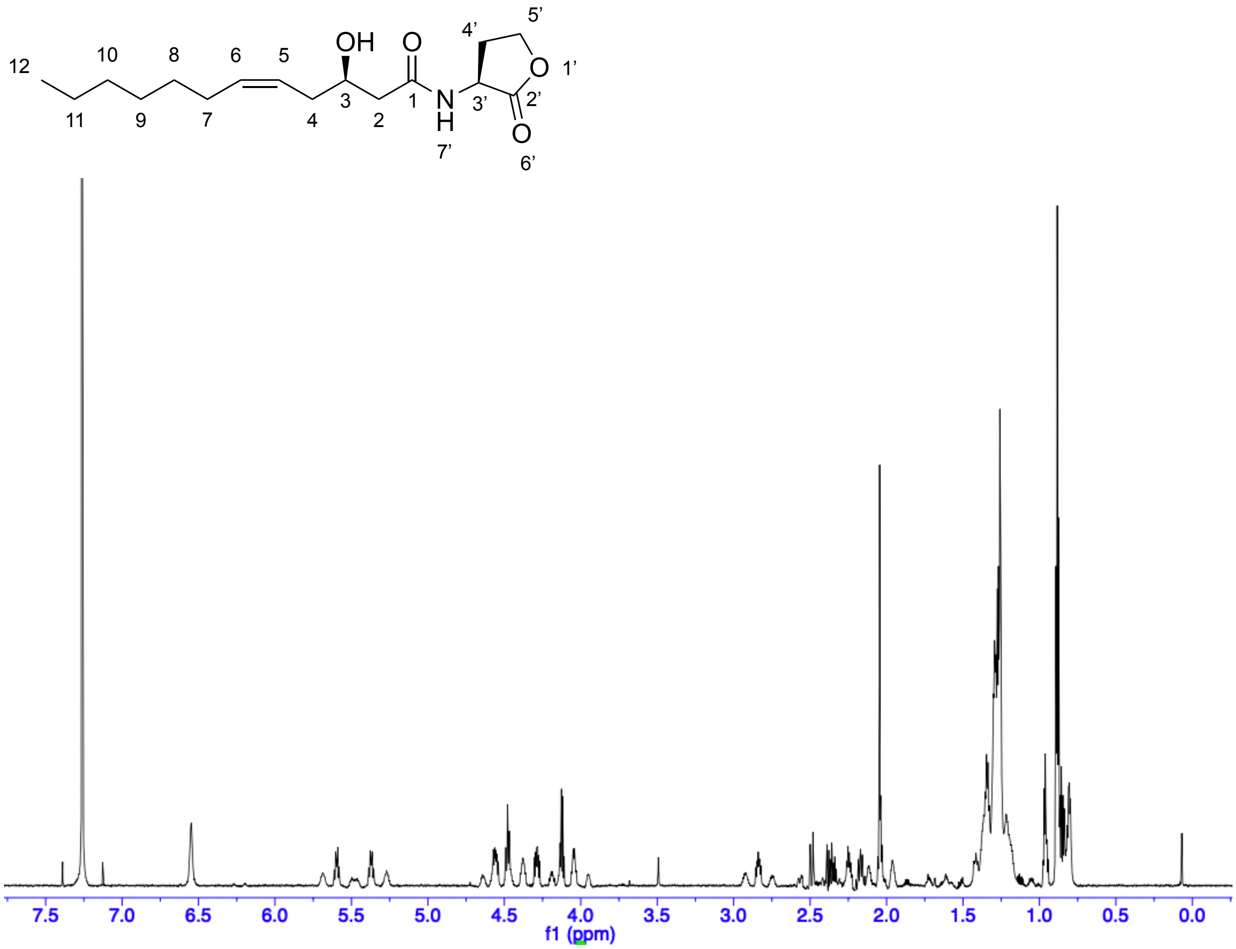

Figure S1. ^{1}H NMR spectrum of *3R*-OH-*5Z*-$C_{12:1}$-HSL in $CDCl_3$ (800 MHz).

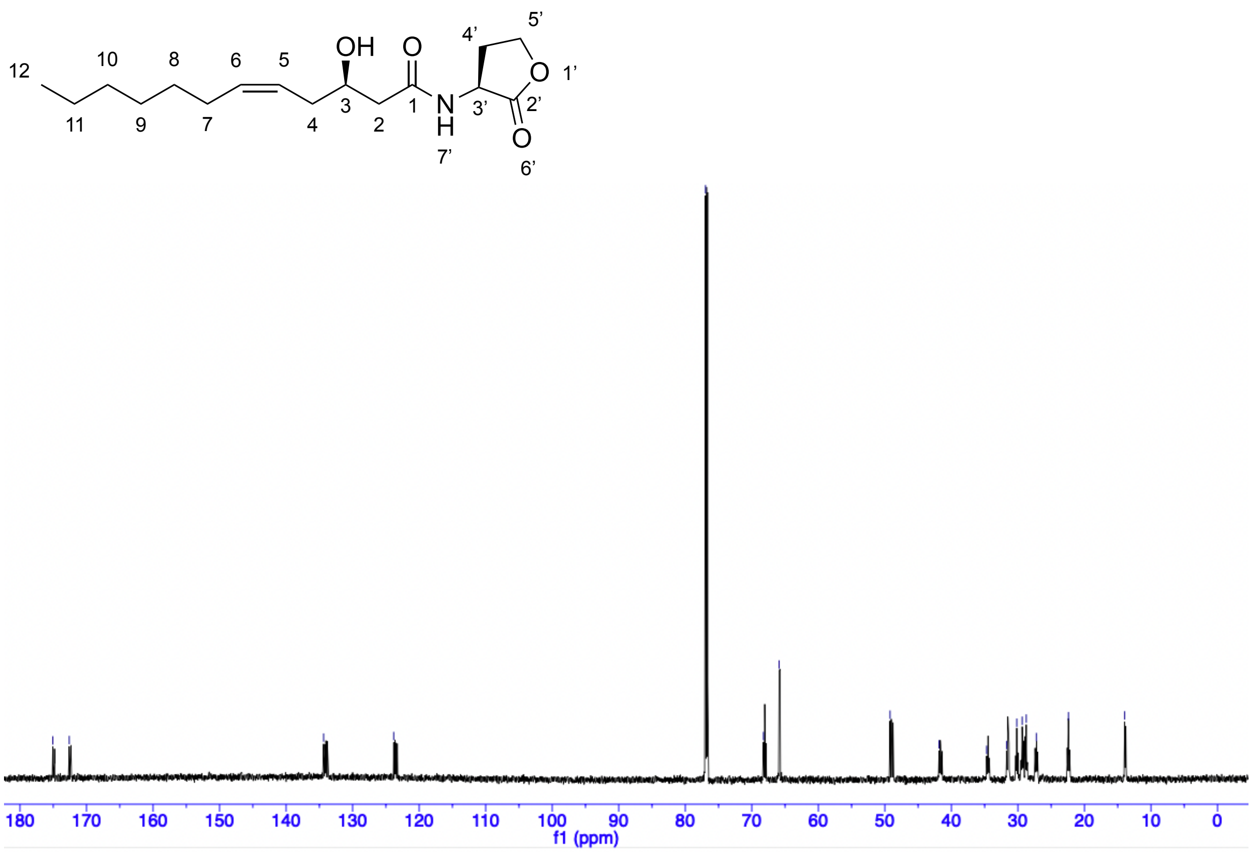

Figure S2. ^{13}C NMR spectrum of *3R*-OH-*5Z*-$C_{12:1}$-HSL in $CDCl_3$ (200 MHz).

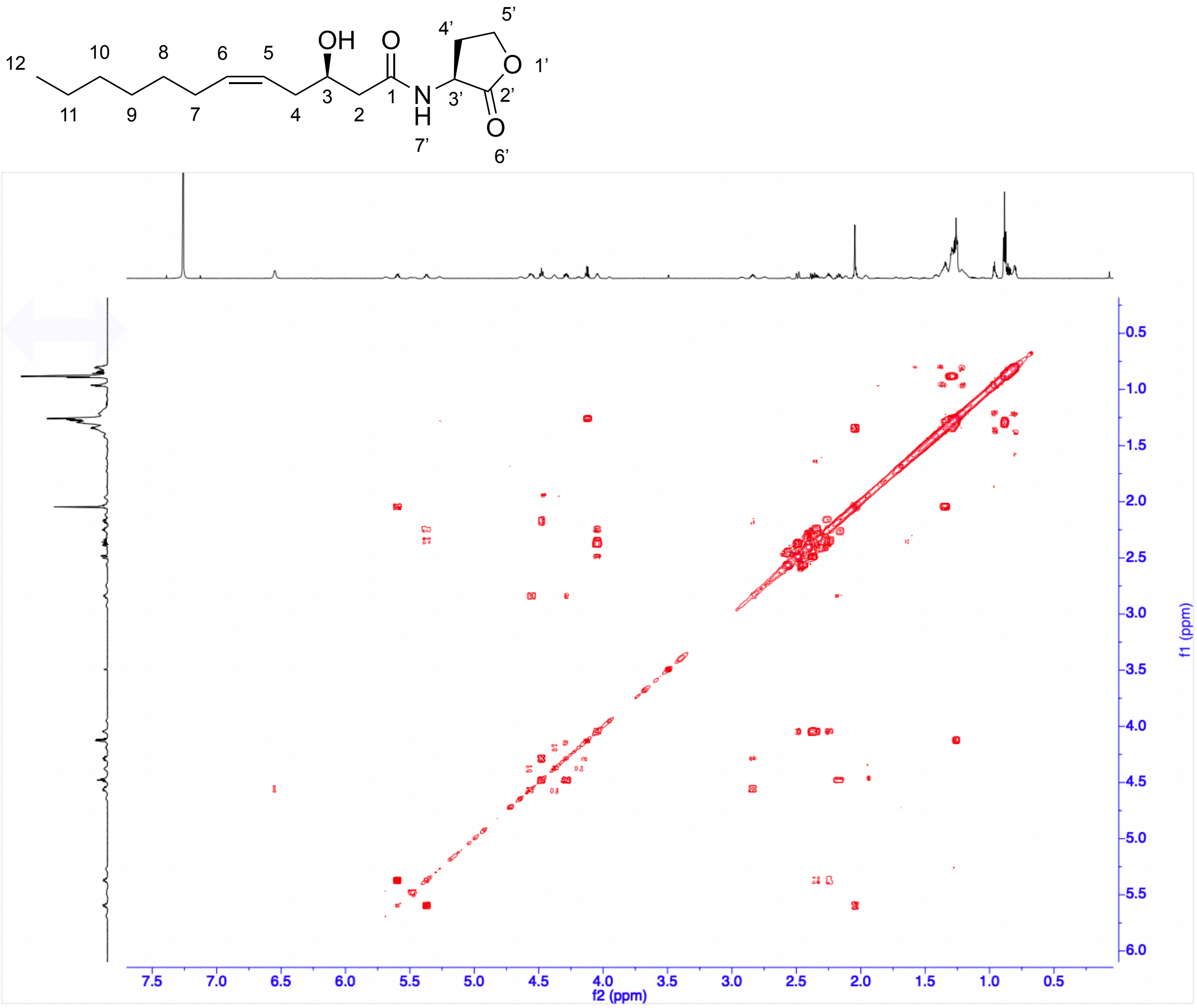

Figure S3. COSY spectrum of *3R*-OH-*5Z*-$C_{12:1}$-HSL in $CDCl_3$ (800 MHz).

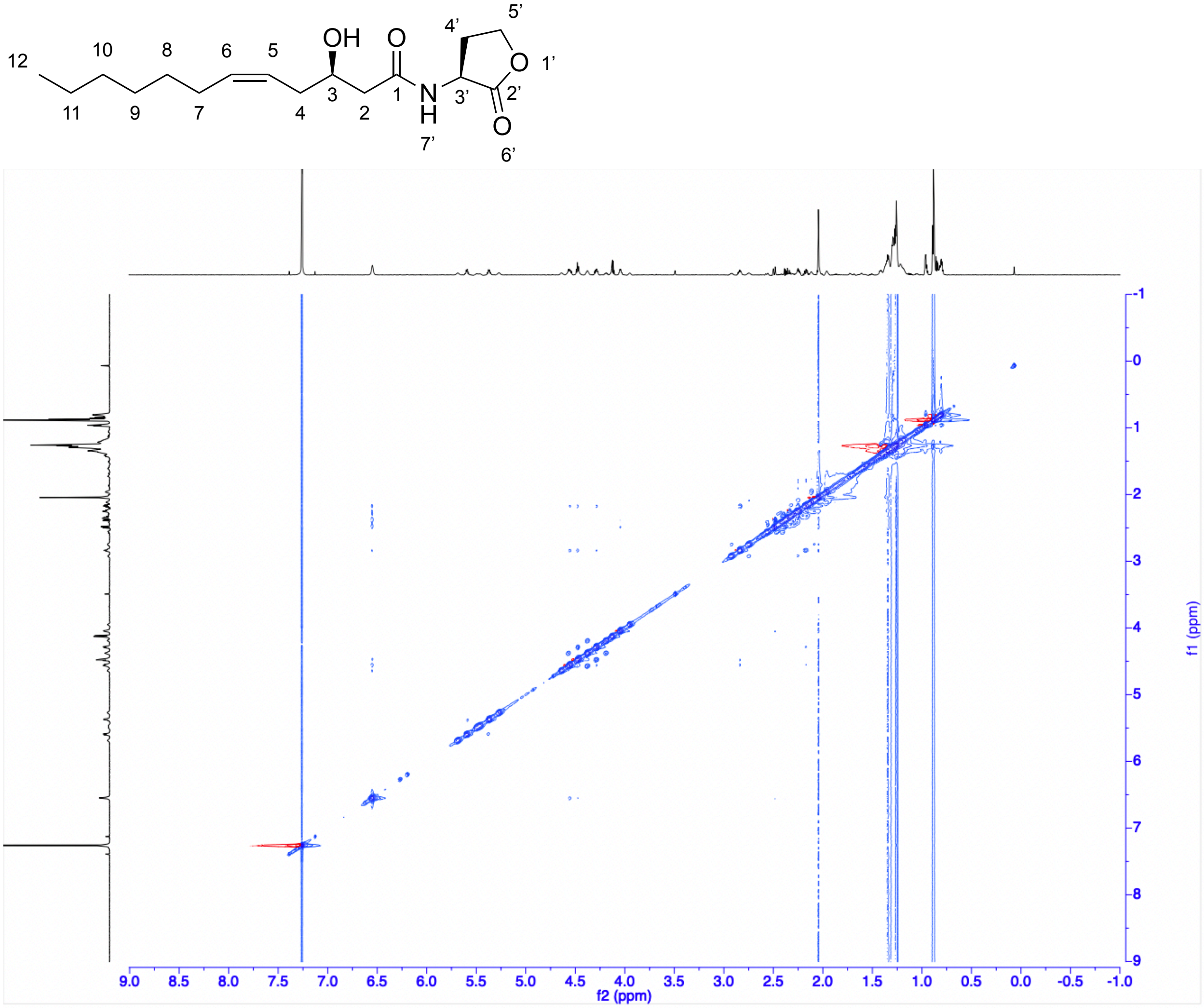

Figure S4. NOESY spectrum of 3*R*-OH-5*Z*-$C_{12:1}$-HSL in $CDCl_3$ (800 MHz).

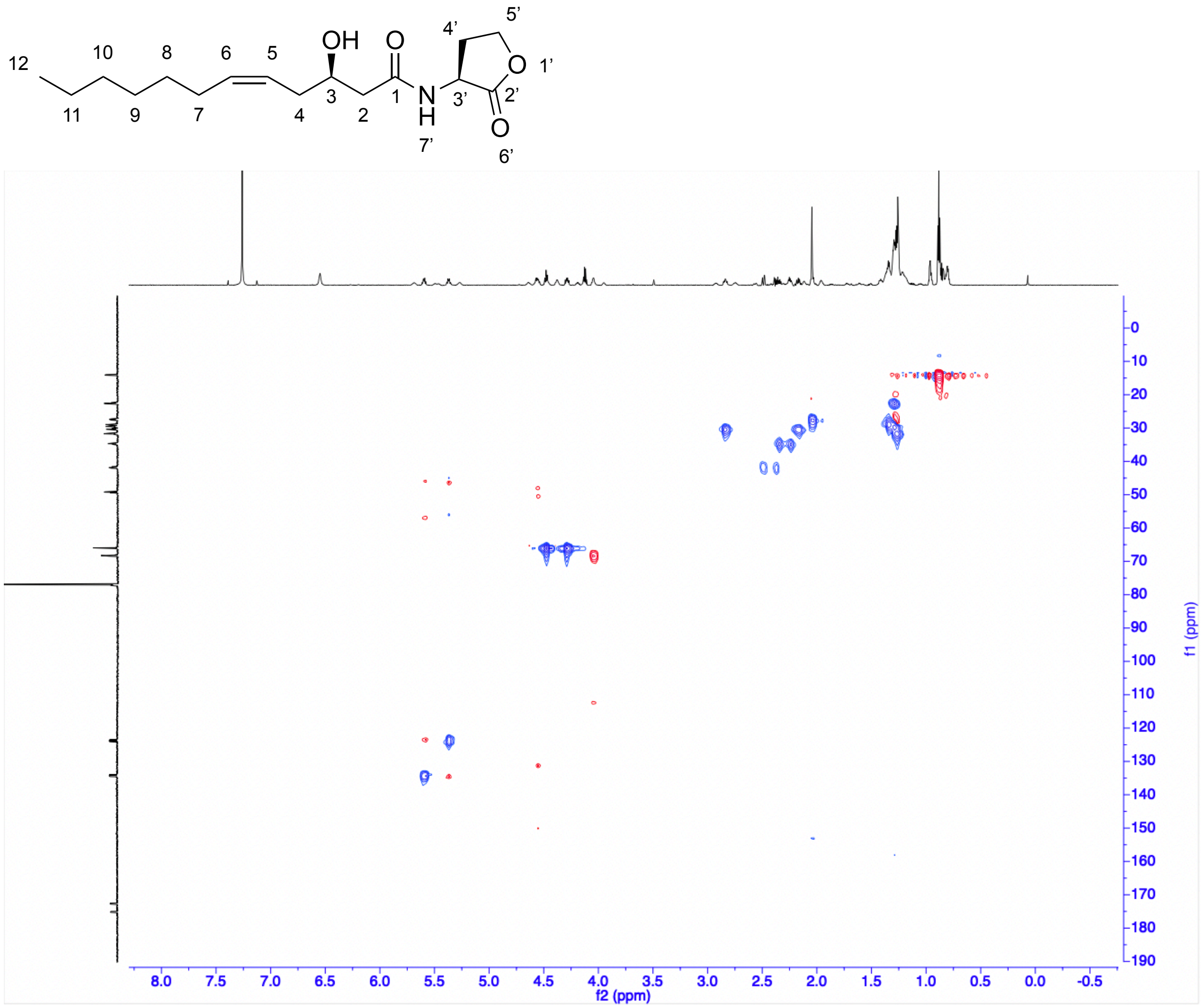

Figure S5. gHSQC spectrum of 3*R*-OH-5*Z*-$C_{12:1}$-HSL in $CDCl_3$ (800 MHz).

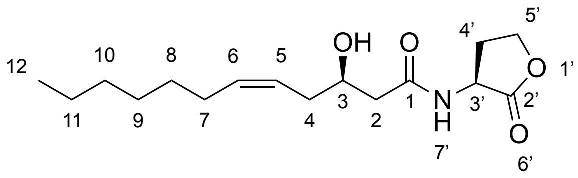

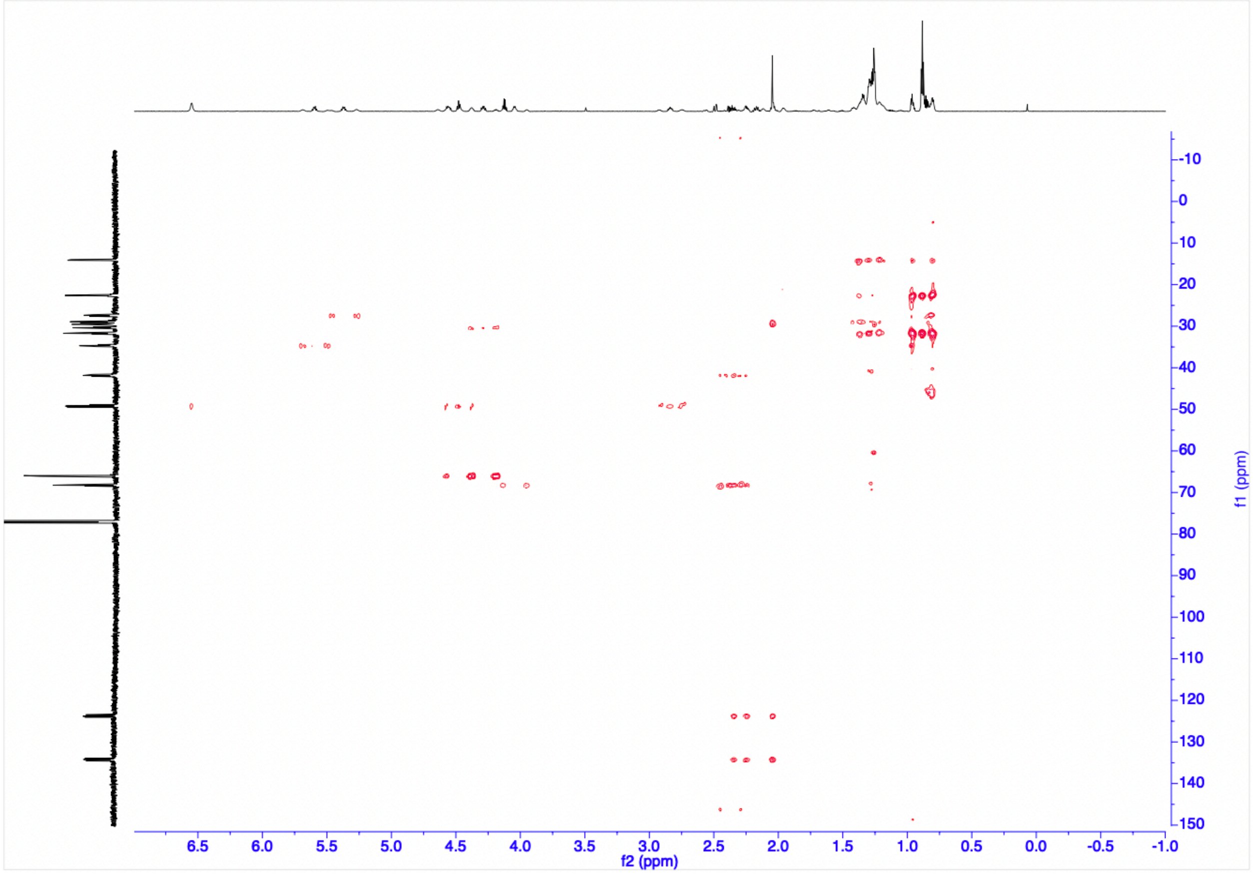

Figure S6. gHMBCAD spectrum of *3R*-OH-5*Z*-$C_{12:1}$-HSL in $CDCl_3$ (800 MHz).

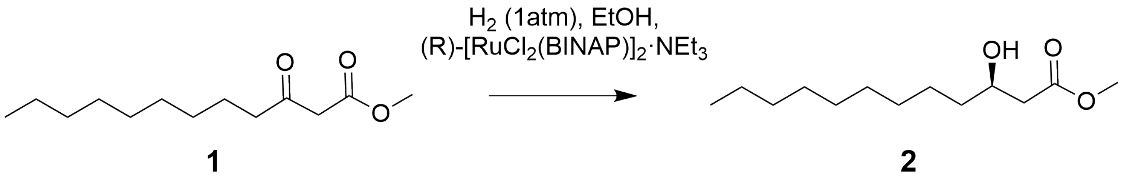

Figure S7. Synthesis of methyl 3*R*-OH-dodecanoate standard.

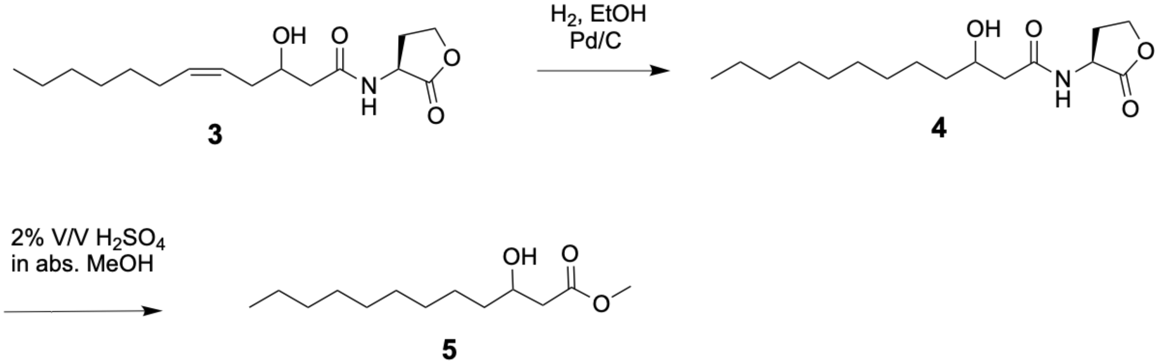

Figure S8. Derivatization of 3*R*-OH-5*Z*-$C_{12:1}$-HSL for chiral GC analysis.

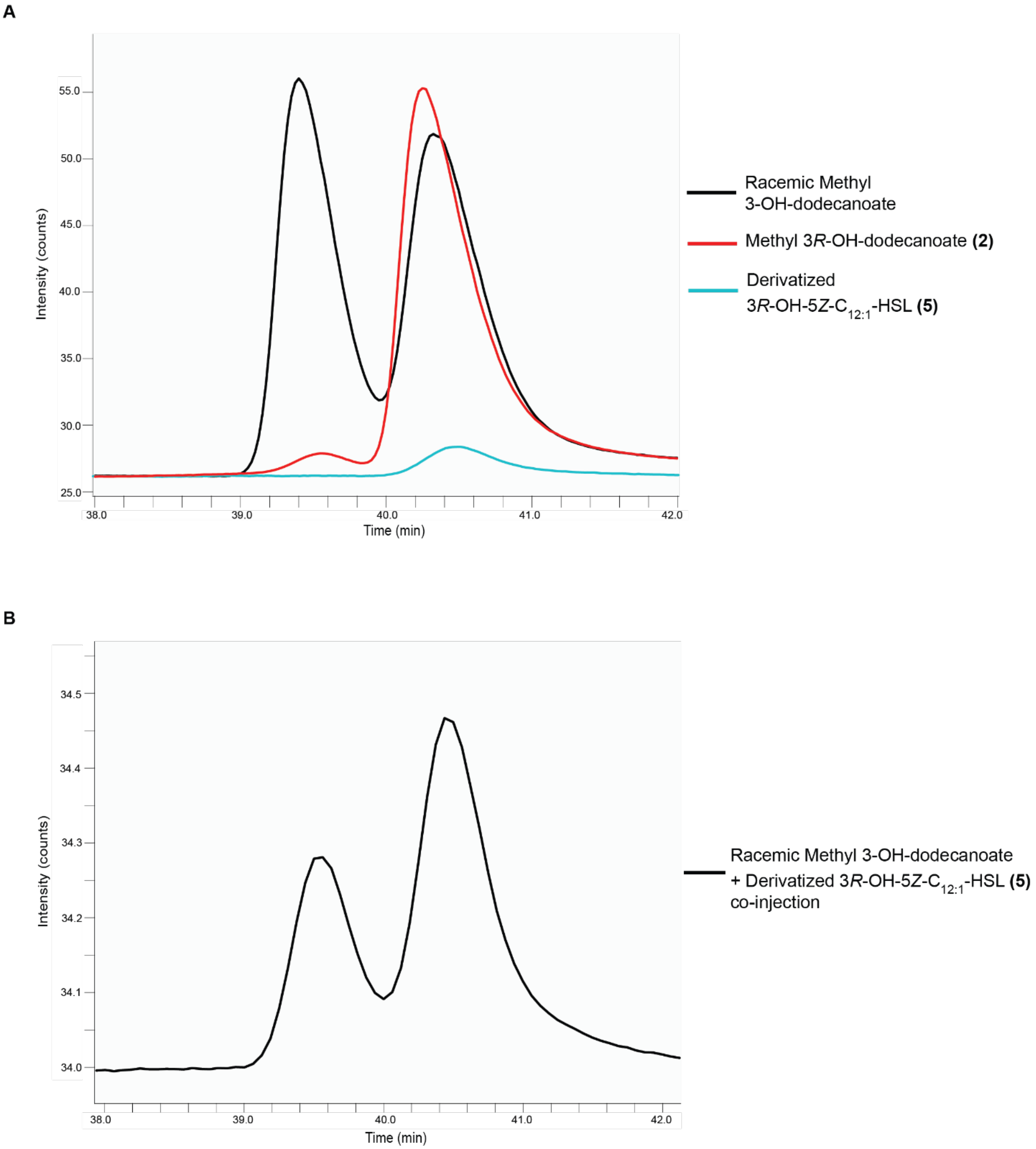

Figure S9. Gas chromatography analysis of derivatized natural 3*R*-OH-5*Z*-$C_{12:1}$-HSL, supporting the determined *R* stereochemistry of the 3-hydroxyl group. (A) GC trace overlays of racemic methyl 3-OH-dodecanoate, methyl 3*R*-OH-dodecanoate and derivatized natural 3*R*-OH-5*Z*-$C_{12:1}$-HSL. (B) Co-injection of racemic methyl 3-OH-dodecanoate and derivatized natural 3*R*-OH-5*Z*-$C_{12:1}$-HSL.

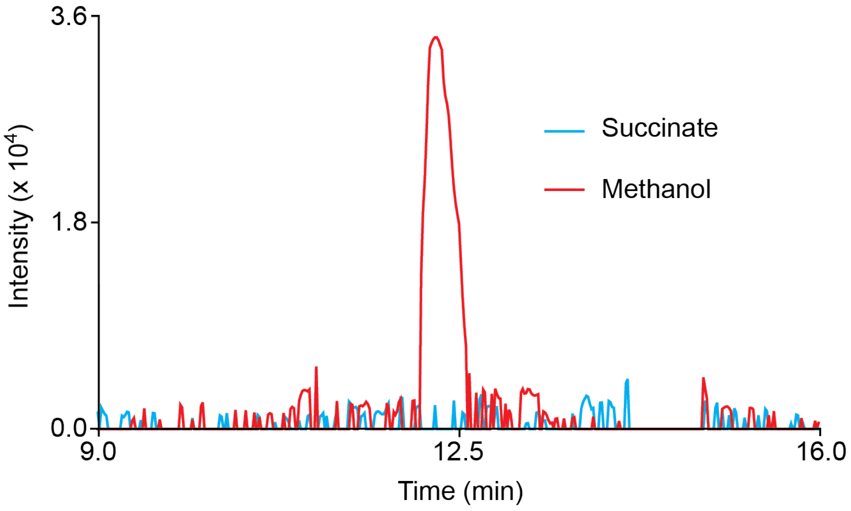

Figure S10. Extracted ion chromatogram of supernatant extracts of DSM5686 grown on the indicated carbon sources for *m/z* 298.2, corresponding to protonated 3*R*-OH-5*Z*-$C_{12:1}$-HSL. Mass tolerance ± 0.5 *m/z*.

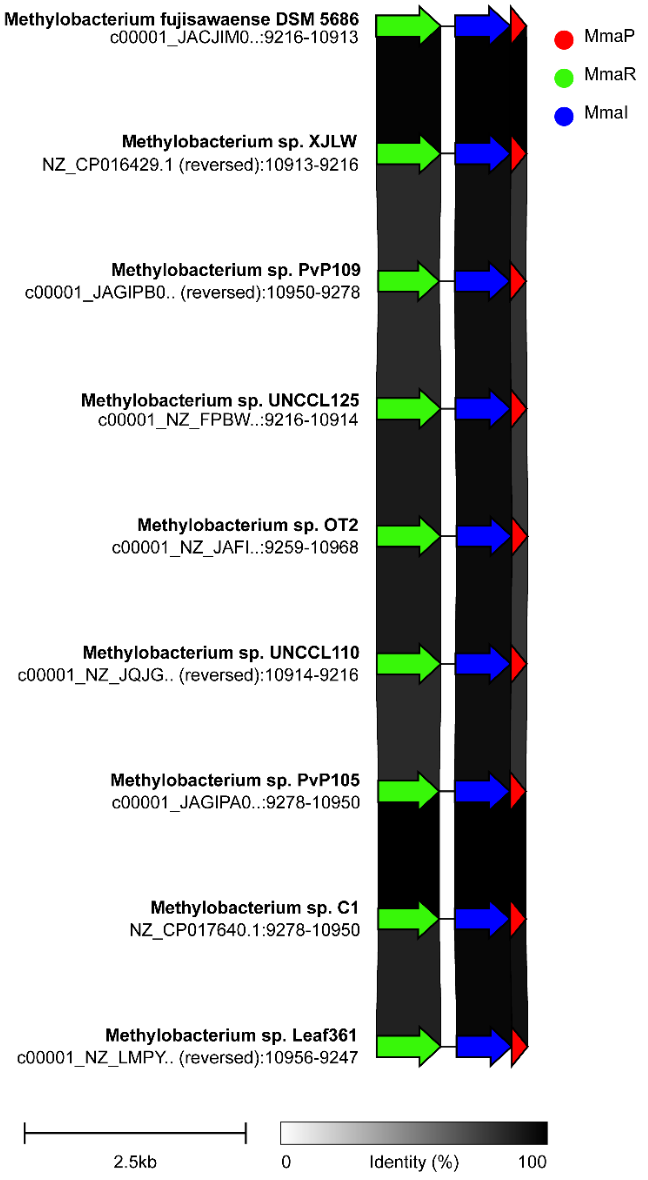

Figure S11. The Mma QS gene neighborhood is highly homologous in different PPFM genomes. Gene neighborhood analysis of *mmaP* in DSM5686 and representative PPFM strains. Analysis was performed using the CAGECAT cblaster and clinker webtools (7). The percent amino acid identities shown are comparing the products of genes to those in strains listed immediately above and below in the figure. Of the strains listed, the minimum percent identities to the three Mma QS genes products in DSM5686 are: *Methylobacterium* sp. PvP109 (91% identical to MmaR and 95% identical to MmaI) and *Methylobacterium* sp. Leaf361 (83% identical to MmaP) The cutoff for a link being shown is 30% identity.

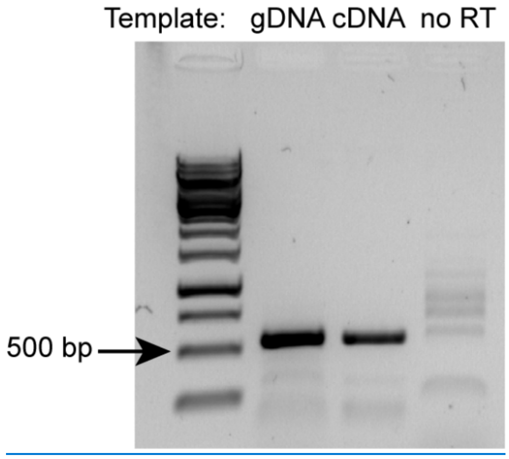

Figure S12. *mmaI* is cotranscribed with *mmaP*. PCR amplification of a genomic region that spans the *mmaI* and *mmaP* open reading frames. The template for PCR was either gDNA, cDNA, or RNA not treated with reverse transcriptase (RT). Expected product size is 526 bp. Product identity was confirmed by Sanger sequencing.

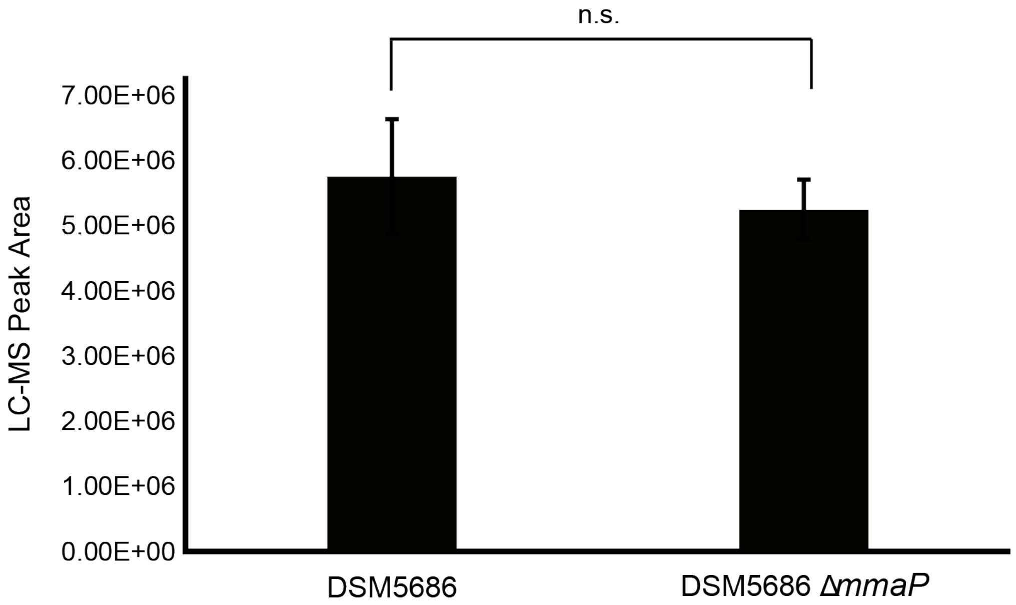

Figure S13. Wild-type DSM5686 and DSM5686Δ*mmaP* produce the same amount of 3*R*-OH-5*Z*-$C_{12:1}$-HSL. The area under the curve of the LC-MS peak of the 3*R*-OH-5*Z*-$C_{12:1}$-HSL feature was quantified. Data show the mean and standard deviation of five technical replicates and are representative of two independent experiments. n.s., not significant (student's t-test, $p < 0.05$).

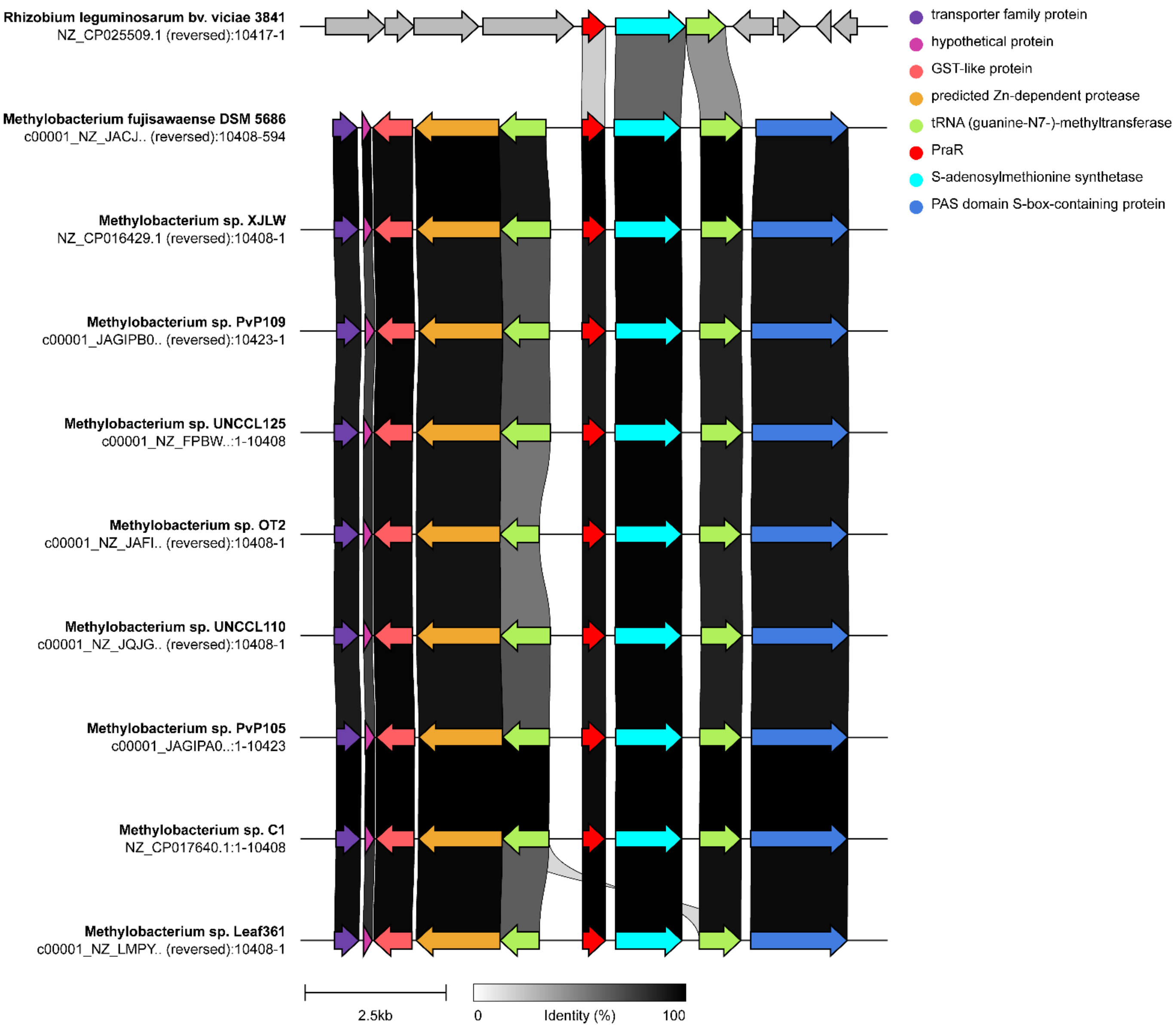

Figure S14. Gene neighborhood analysis of *praR* in *Rhizobium leguminosarum* bv. viciae 3841, DSM5686 and representative *Methylobacterium* strains. Analysis was performed using the CAGECAT cblaster and clinker webtools (7). The cutoff for a link being shown is 30% amino acid identity.

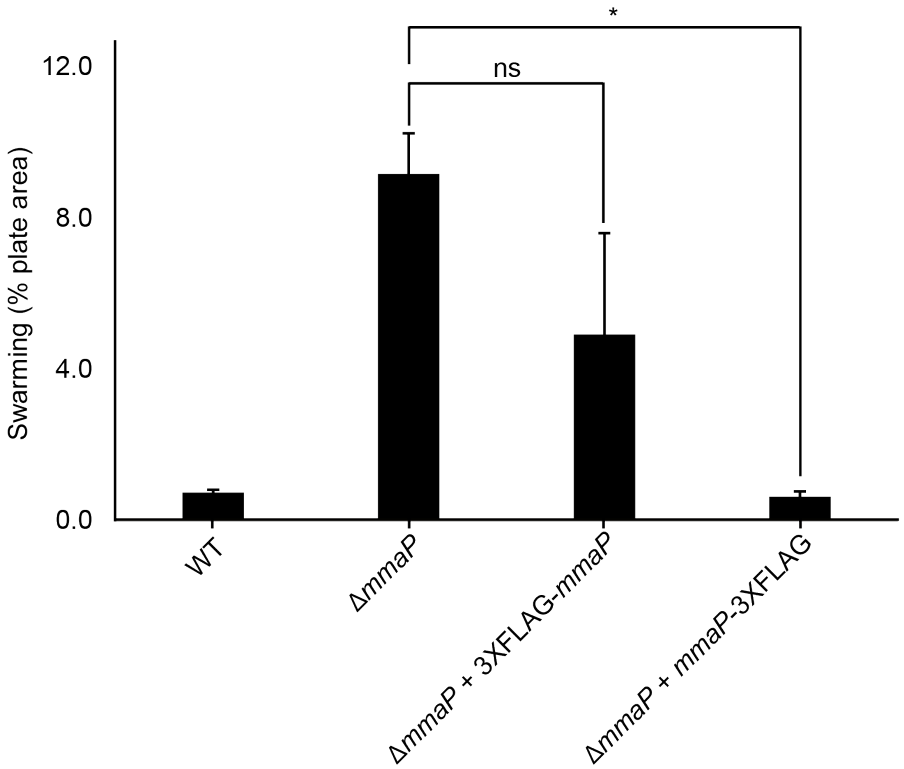

Figure S15. A C-terminally 3XFLAG-tagged MmaP is still capable of repressing swarming in DSM5686. Data show the mean and standard deviation of three plates and are representative of two independent experiments. Means were compared to WT using a 1-way ANOVA with Tukey's post-hoc test. *, P <0.001; ns, not significant.

SUPPLEMENTARY TABLES

Table S1. MS/MS peak list for *m/z* 298 feature compared to commercial 3-oxo-C_{12}-HSL standard. The 15 most intense signals are shown for each sample.

**3-oxo-C12-HSL**		**88A**		**JCM2831**		**DSM 5686**		**AWP314**	
m/z	**Intensity**	***m/z***	**Intensity**	***m/z***	**Intensity**	***m/z***	**Intensity**	***m/z***	**Intensity**
302.3053	1.95E+05	102.0564	4.02E+05	102.0566	1.99E+05	102.0569	1.33E+06	102.0565	1.51E+06
298.2013	5.53E+04	298.2016	3.22E+05	298.2019	1.50E+05	298.2021	1.08E+06	298.2018	1.24E+06
197.1533	4.60E+04	280.191	1.29E+05	280.1914	6.02E+04	280.1914	3.75E+05	280.191	4.13E+05
102.0565	4.01E+04	179.1432	6.63E+04	95.0876	3.19E+04	95.0878	2.38E+05	95.0875	2.76E+05
299.205	1.03E+04	299.2048	6.45E+04	299.2049	3.10E+04	179.1434	2.02E+05	299.2049	2.36E+05
155.1429	8.69E+03	155.1432	5.43E+04	179.1433	3.10E+04	137.1332	1.94E+05	137.1328	2.32E+05
198.157	5.76E+03	95.0873	5.18E+04	161.1327	2.46E+04	155.1436	1.83E+05	179.1432	2.27E+05
284.2951	5.42E+03	137.1328	5.03E+04	137.1331	2.46E+04	161.1329	1.60E+05	155.1432	2.13E+05
240.196	4.44E+03	161.1325	4.83E+04	155.1435	2.45E+04	81.0729	1.41E+05	161.1326	1.76E+05
270.2073	3.73E+03	81.0724	3.05E+04	81.0727	1.69E+04	281.1947	7.23E+04	81.0725	1.69E+05
98.0614	2.93E+03	281.1944	2.47E+04	302.3058	1.34E+04	103.06	7.12E+04	281.1943	8.05E+04
302.2688	2.41E+03	103.0596	2.21E+04	281.1949	1.25E+04	119.0868	6.19E+04	103.0597	7.98E+04
74.0632	2.29E+03	252.1961	1.63E+04	103.0597	1.04E+04	252.1963	5.31E+04	119.0862	6.96E+04
280.1902	2.22E+03	234.1857	1.62E+04	119.0865	9.68E+03	109.1026	5.24E+04	109.1024	5.95E+04
252.1969	2.17E+03	119.0863	1.48E+04	74.0632	8.12E+03	74.0635	5.17E+04	74.0633	5.61E+04

Table S2. NMR assignments for *3R*-OH-*5Z*-$C_{12:1}$-HSL in $CDCl_3$

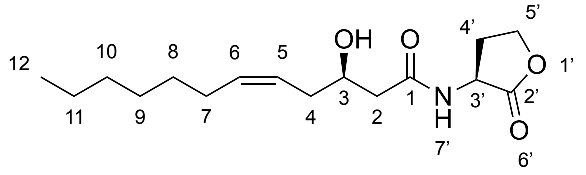

Position	δ_c, type	δ_H (*J* in Hz)	HMBC
5'	65.9, CH_2	4.28, 4.48, m	2', 3', 4'
4'	29.4, CH_2	2.84, m	2', 3', 5'
3'	49.2, CH	4.56, dd (6.7) (1.7)	2', 4', 5'
2'	179.3, C		3', 4', 5'
1	176.5, C		2, 3
2	41.9 CH_2	2.38, 2.50, dd (5.8)	1, 3
3	68.3, CH	4.04, tt (7.7)	1, 2, 4
4	34.7, CH_2	2.25, 2.36, dd (6.9)	2, 5, 6
5	127.8, CH	5.59, (8.9)	4, 6, 7
6	138.2, CH	5.36, (8.9)	4, 5, 7, 8
7	27.2, CH_2	2.17, q (8.9)	5, 6, 8, 9
8	28.8, CH_2	2.05, tt (7.2)	6, 7, 9, 10, 11
9	31.7, CH_2	1.34, m	7, 8, 10, 11, 12
10	30.2, CH_2	1.28, m	8, 9, 11
11	22.4, CH_2	1.26, m	8, 9, 10, 12
12	14.0, CH_3	0.88, t (7.0)	9, 10, 11
NH (7')		6.55	

Table S3. Marfey's analysis of HSL portion of *3R*-OH-*5Z*-$C_{12:1}$-HSL.

**Sample**	**Retention time (min)**
Marfey's derivatized *D*-HSL standard (*m/z* 372)	13.6
Marfey's derivatized *L*-HSL standard (*m/z* 372)	15.2
Marfey's derivatized *3R*-OH-*5Z*-$C_{12:1}$-HSL hydrolysate (*m/z* 372)	15.4

Table S4. Strains used in this study.

Strain	Puri Lab Strain Collection Number	Description[a]	Reference
E. coli TOP10	EAWP2	F– *mcrA* Δ(*mrr-hsdRMS-mcrBC*) Φ80*lacZ*ΔM15 Δ*lacX74 recA1 araD139* Δ(*ara leu*) 7697 *galU galK rpsL* (Str^R) *endA1 nupG*	Invitrogen
E. coli S17-1 λpir	EAWP3	Donor strain. Tp^R Sm^R *recA thi pro hsd*(r^-m^+) RP4-2-Tc::Mu::Km Tn7 λ*pir*	(8)
E. coli BL21 (DE3) star + pAWP548+pAWP554	EAWP359	N-terminal His $praR_{DSM5686}$ and $mmaP_{DSM5686}$-3XFLAG driven by constitutive nptII promoter	This study
E. coli BL21 (DE3) star + pAWP548+pAWP555	EAWP360	C-terminal His $praR_{DSM5686}$ and $mmaP_{DSM5686}$-3XFLAG driven by constitutive nptII promoter	This study
E. coli BL21 (DE3) star + pAWP548	EAWP375	$mmaP_{DSM5686}$-3XFLAG driven by constitutive nptII promoter in pACYC vector	This study
Methylobacterium sp. strain 88A	AWP105	Pink pigmented facultative methylotroph.	(9)
M. radiotolerans JCM2831	AWP120	Pink pigmented facultative methylotroph. Source of $mmaI_{JCM2831}$ gene.	(10)
M. extorquens AWP227	AWP227	Heterologous expression strain. Derivative of *M. extorquens* PA1. Δ(*celABC*-Mext_1370) Δ*mlaRI*	(1)
M. fujisawaense DSM5686	AWP269	Pink pigmented facultative methylotroph.	(11)
M. extorquens AWP227 + pAWP417	AWP314	Heterologous expression of $mmaI_{JCM2831}$	This study
M. extorquens AWP348	AWP348	Insertion mutant containing $mmaR_{DSM5686}$ downstream of the *mlaR* promoter in AWP227. Δ(*celABC*-Mext_1370) Δ*mlaRI::mmaR*$_{DSM5686}$	This study
M. extorquens AWP348 + pAWP492	AWP370	$mmaR_{DSM5686}$ reporter strain.	This study
M. fujisawaense DSM5686 AWP373	AWP373	DSM5686 Δ*mmaP*	This study
M. fujisawaense DSM5686 AWP374	AWP374	DSM5686 Δ$mmaI_{DSM5686}$	This study

AWP373 + pAWP504	AWP378	DSM5686 Δ*mmaP* + 3XFLAG-*mmaP*$_{DSM5686}$ under *mmaI* promoter	This study
AWP373 + pAWP505	AWP379	DSM5686 Δ*mmaP* + *mmaP*$_{DSM5686}$-3XFLAG under *mmaI* promoter	This study
AWP373 + pAWP506	AWP380	DSM5686 Δ*mmaP* + *mmaP*$_{DSM5686}$ under *mmaI* promoter	This study
M. fujisawaense 5686 AWP389	AWP389	DSM5686 Δ*praR*	This study
M. fujisawaense 5686 AWP390	AWP390	DSM5686 Δ*mmaP* Δ*praR*	This study
AWP389+pAWP547	AWP423	Complement of *praR*$_{DSM5686}$ under native promoter in AWP389	This study
AWP390+pAWP547	AWP424	Complement of *praR*$_{DSM5686}$ under native promoter in AWP390	This study

[a]IMG/M gene locus tags: Δ*mlaRI*$_{PA1}$, Mext_4513-4; *mmaR*$_{DSM5686}$, Ga0373205_3344; *mmaI*$_{DSM5686}$, Ga0373205_3345; *mmaP*$_{DSM5686}$, Ga0373204_3346; *mmaI*$_{JCM2831}$, Mrad2831_5763; *dnaG*$_{PA1}$, Mext_0611; *praR*$_{DSM5686}$, Ga0373204_957.

Table S5. Plasmids used in this study.

Plasmid	Puri Lab Plasmid Collection Number	Description[a]	Reference
pAWP78	pAWP78	IncP-based expression vector.	
pAWP227	pAWP227	pCM433kanT containing flanks to knock out *mlaRI* in PA1.	(1)
pAWP274	pAWP274	Inserting $mmaR_{DSM5686}$ into AWP227.	This study.
pCM433kanT	pAWP285	Sucrose counterselection vector for creating unmarked deletion mutants.	(12)
pMRE-Tn7-135	pAWP398	Source of *mScarlet* gene.	(13)
pAWP417	pAWP417	Expressing $mmaI_{JCM2831}$ gene under the $dnaG_{PA1}$ promoter (400 bp upstream sequence).	This study
pAWP429	pAWP429	pCM433kanT containing flanks to knock out the *mmaI* gene in DSM5686.	This study
pAWP492	pAWP492	Expressing mScarlet under the $mmaI_{DSM5686}$ promoter (400 bp upstream sequence).	This study
pAWP504	pAWP504	3XFLAG-$mmaP_{DSM5686}$ (*E. coli* optimized) under MmaI promoter	This study
pAWP505	pAWP505	$mmaP_{DSM5686}$ (*E. coli* optimized)-3XFLAG under *mmaI* promoter	This study
pAWP506	pAWP506	$mmaP_{DSM5686}$ (*E. coli* optimized) under *mmaI* promoter, no FLAG tag	This study
pAWP516	pAWP516	KO of $praR_{DSM5686}$	This study
pAWP547	pAWP547	$praR_{DSM5686}$ under native promoter	This study
pAWP548	pAWP548	$mmaP_{DSM5686}$-3XFLAG driven by constitutive *nptII* promoter in pACYC vector	This study
pAWP554	pAWP554	$praR_{DSM5686}$ (*E. coli* optimized) N-terminal His tag in pET28(a) vector	This study
pAWP555	pAWP555	$praR_{DSM5686}$ (*E. coli* optimized) C-terminal His tag pET28(a) vector	This study

[a]IMG/M gene locus tags: $\Delta mlaRI_{PA1}$, Mext_4513-4; $mmaR_{DSM5686}$, Ga0373205_3344; $mmaI_{DSM5686}$, Ga0373205_3345; $mmaP_{DSM5686}$, Ga0373204_3346; $mmaI_{JCM2831}$, Mrad2831_5763; $dnaG_{PA1}$, Mext_0611; $praR_{DSM5686}$, Ga0373204_957.

Table S6. Primers used in this study. Homology regions used for Gibson Assembly are bolded.

Primer Name	Sequence (5' to 3')	Description[a]
oAWP186_ 433KTV1_fwd	ATGTGCAGGTTGTCGGTGTC	For amplifying the pCM433kanT backbone. oAWP186 and 160 were used to amplify one piece, and oAWP159 and 187 were used to amplify the other.
oAWP160_ 433KTV1_rev	**ATAAAGGTGAATCCCATAGGGCAGGAGCTA**TAATCTCGAGTCCCGTCAAG	
oAWP159_433KTV2_fwd	TAGCTCCTGCCCTATGGGAT	
oAWP187_433KTV2_rev	TGGTAACTGTCAGACCAAGTTTACTC	
oAWP259_78V_fwd1	TTGTCGGGAAGATGCGTGAT	For amplifying the pAWP78 backbone.
oAWP254_78V_rev1	CAGCTCACTCAAAGGCGGTA	
oAWP994_PdnaG_fwd	**AGCGCGTACTCCGTCCCCCGAACGTTGCAT**GGGGACTCTGCTGGAAGCGG	For amplifying the *dnaG*$_{PA1}$ promoter (400 bp upstream sequence) for heterologous expression of *mmaI*$_{JCM2831}$.
oAWP995_PdnaG_rev	**TCCACGCGATCCGCTTCCAGCAGAGTCCCC**ATGATCCATGTCGTGACTGC	
oAWP1055_fwd	CATGGCCCGGCAAAATCATC	For opening pAWP227 between the up and down flanks to insert *mmaR*$_{DSM5686}$. oAWP1055 was used with oAWP160 and oAWP1056 was used with oAWP159.
oAWP1056_rev	CGGACGCGGCGACGGGTGCG	
oAWP1060_274I_fwd	**ATGAGAGTGCGATGATTTTGCCGGGCCATG**CCGCATTCGAAGCACCTGGA	For amplifying the *mmaR*$_{DSM5686}$ to insert between the up and down flanks of pAWP227.
oAWP1061_274I_rev	**ACGGATGCGGCGCACCCGTCGCCGCGTCCG**CTACGTGATCAGGCCGGCAC	
oAWP1086_417I_fwd	**GAAGGATCAGATCACGCATCTTCCCGACAA**ATGATCCATATCGTCACACCCGCC	For amplifying *mmaI*$_{JCM2831}$.
oAWP1087_417I_rev	**TCCACGCGATCCGCTTCCAGCAGAGTCCCC**TCAGGCCACCAGGTAAGCGGGTT	
oAWP1448_429U_fwd1	**TTTTGCCGGGCCATG**TTCAAACGGCAGAGCTTTCC	For amplifying flanks to knock out *mmaI* in DSM5686.
oAWP1449_429U_rev1	**GGCGAGCCGGGCGAA**CACGAAGTAACCTCACGTTG	

oAWP1450_429D_fwd1	**CGTGCATCACGACACCGACAACCTGCACAT**AACTCGGTCCTGGTCTCCGA	
oAWP1446_429D_rev1	**ATAACCGTATTACCGCCTTTGAGTGAGCTG**AGTACCCGGTCTACCTCTCG	
oAWP1535_450I2_fwd	ATGGTGAGCAAGGGCGAGG	For amplifying *mScarlet* from pMRE-Tn7-135.
oAWP1504_450I2_rev	**GAAGGATCAGATCACGCATCTTCCCGACAA**CTTGTACAGCTCGTCCATGCC	
oAWP1617 fwd 5686 upstream I gene	**ATAACCGTATTACCGCCTTTGAGTGAGCTG**CAACTCCTGCTGATGAACAGC	For amplifying the *mmaI*$_{DSM5686}$ promoter (400 bp upstream sequence).
oAWP1618 rev 5686 upstream I gene	**ATAACCGTATTACCGCCTTTGAGTGAGCTG**CAACTCCTGCTGATGAACAGC	
oAWP1617 fwd 5686 upstream I gene	**ATAACCGTATTACCGCCTTTGAGTGAGCTG**CAACTCCTGCTGATGAACAGC	For amplifying the *mmaI*$_{DSM5686}$ promoter (400 bp upstream sequence). For use in pAWP492
oAWP1635_pAWP492_luxI_rev	GAAGTAACCTCACGTTGGAT	
oAWP1636_pAWP486_3xFLAGpep_fwd	**ATCCAACGTGAGGTTACTTCAT**GGACTACAAAGACCATGA	For amplifying *mmaP*$_{DSM5686}$ with N-terminal 3xFLAG tag
oAWP1608_486I_rev	**ATCACGCATCTTCCCGACAA**TTATGCACCGCCCGCACGTT	
oAWP1637_pAWP487_pep3XFLAG_fwd	**ATCCAACGTGAGGTTACTTC**ATGTTTGATAGCAAAGATGT	For amplifying *mmaP*$_{DSM5686}$ with C-terminal 3xFLAG tag
oAWP1610_487I_rev	**ATCACGCATCTTCCCGACAA**TTACTTGTCATCGTCATCCT	
oAWP1639_pAWP495_5686pep_fwd	**ATCCAACGTGAGGTTACTTC**ATGTTCGACTCTAAGGACGTGGCTTTGG	For amplifying *mmaP*$_{DSM5686}$
oAWP1608_486I_rev	**ATCACGCATCTTCCCGACAA**TTATGCACCGCCCGCACGTT	
oAWP1684_516U_fwd	**CTGAATTCAGCTGTACAATTGGTAC**CCTCCACGACGACGAAATGC	For amplifying upstream region of *praR*$_{DSM5686}$
oAWP1685_516U_rev	**GCCGATCTTGCTCTC**CATGAAGTGCTCGTCCACCC	
oAWP1686_516D_fwd	**GACGAGCACTTCATG**GAGAGCAAGATCGGCGGTTG	For amplifying downstream region of *praR*$_{DSM5686}$
oAWP1687_516D_rev	**CATCGGCTGGATCCTCTAGTGAGCT**GACCGCCGATCACGAACTTG	
oAWP1770_546I_rev	**GAAGGATCAGATCACGCATCTTCCCGACAA**TCAACCGCCGATCTTGC	For amplifying *praR*$_{DSM5686}$ and 400bp upstream region
oAWP1771_547I_fwd	**ATAACCGTATTACCGCCTTTGAGTGAGCTG**GCGGATCCGCTCAAAG	
oAWP1772_548I1_fwd	**TTTATCTCTTCAAATGTAGCACCTGAAGTC**ATTGACCCATAAACTGCCAG	For amplifying *nptII* 400bp promoter region
oAWP1773_548I1_rev	**AGCGCGTCAAGAGCTACATCTTTGCTATCAAA**CATTTTTTCTTCCTCCACTAGTATCC	
oAWP1774_548I2_fwd	**AGAGACAGGATACTAGTGGAGGAAGAAAAA**ATGTTTGATAGCAAAGATGT	

oAWP1775_548I2_rev	**CCGCTCATGGCGTTGACTCTCAGTCATAGT**TTACTTGTCATCGTCATC	For amplifying *mmaP*$_{DSM5686}$ with C term 3xFLAG tag
oAWP1781_5686RTPCR_fwd	GCATCTTCTGTCCGATGTCC	For amplifying across *mmaI*$_{DSM5686}$ and *mmaP*$_{DSM5686}$ open reading frames
oAWP1782_5686RTPCR_rev	TCGAGAAGCAGGAGGTCGAT	

[a]IMG/M gene locus tags: Δ*mlaRI*$_{PA1}$, Mext_4513-4; *mmaR*$_{DSM5686}$, Ga0373205_3344; *mmaI*$_{DSM5686}$, Ga0373205_3345; *mmaP*$_{DSM5686}$, Ga0373204_3346; *mmaI*$_{JCM2831}$, Mrad2831_5763; *dnaG*$_{PA1}$, Mext_0611; *praR*$_{DSM5686}$, Ga0373204_957.

SUPPLEMENTARY REFERENCES

1. Whittenbury R, Phillips KC, Wilkinson JF. 1970. Enrichment, isolation and some properties of methane-utilizing bacteria. J Gen Microbiol 61:205–218 https://doi.org/10.1099/00221287-61-2-205.

2. Pluskal T, Castillo S, Villar-Briones A, Orešič M. 2010. MZmine 2: Modular framework for processing, visualizing, and analyzing mass spectrometry-based molecular profile data. BMC Bioinformatics 11:395 https://doi.org/10.1186/1471-2105-11-395.

3. Myers OD, Sumner SJ, Li S, Barnes S, Du X. 2017. One Step Forward for Reducing False Positive and False Negative Compound Identifications from Mass Spectrometry Metabolomics Data: New Algorithms for Constructing Extracted Ion Chromatograms and Detecting Chromatographic Peaks. Anal Chem 89:8696–8703 https://doi.org/10.1021/acs.analchem.7b00947.

4. Gibson DG, Young L, Chuang R-Y, Venter JC, Hutchison CA, Smith HO. 2009. Enzymatic assembly of DNA molecules up to several hundred kilobases. Nat Methods 6:343–345 https://doi.org/10.1038/nmeth.1318.

5. Taber DF, Silverberg LJ. 1991. Enantioselective reduction of β-keto esters. Tetrahedron Lett 32:4227–4230 https://doi.org/10.1016/S0040-4039(00)92134-8.

6. Thiel V, Kunze B, Verma P, Wagner-Döbler I, Schulz S. 2009. New Structural Variants of Homoserine Lactones in Bacteria. ChemBioChem 10:1861–1868 https://doi.org/10.1002/cbic.200900126.

7. Gilchrist CLM, Chooi Y-H. 2021. clinker & clustermap.js: automatic generation of gene cluster comparison figures. Bioinformatics 37:2473–2475 https://doi.org/10.1093/bioinformatics/btab007.

8. Simon R, Priefer U, Pühler A. 1983. A Broad Host Range Mobilization System for In Vivo Genetic Engineering: Transposon Mutagenesis in Gram Negative Bacteria. Nat Biotechnol 1:784–791 https://doi.org/10.1038/nbt1183-784.

9. Beck DAC, McTaggart TL, Setboonsarng U, Vorobev A, Goodwin L, Shapiro N, Woyke T, Kalyuzhnaya MG, Lidstrom ME, Chistoserdova L. 2015. Multiphyletic origins of methylotrophy in Alphaproteobacteria, exemplified by comparative genomics of Lake Washington isolates. Environ Microbiol 17:547–554 https://doi.org/10.1111/1462-2920.12736.

10. Green PN, Bousfield IJ. 1983. Emendation of *Methylobacterium* Patt, Cole, and Hanson 1976; *Methylobacterium rhodinum* (Heumann 1962) comb. nov. corrig.; *Methylobacterium radiotolerans* (Ito and Iizuka 1971) comb. nov. corrig.; and *Methylobacterium mesophilicum* (Austin and Goodfellow 1979) comb. nov. Int J Syst Evol Microbiol 33:875–877 https://doi.org/10.1099/00207713-33-4-875.

11. Green PN, Bousfield IJ, Hood D. 1988. Three New *Methylobacterium* Species: *M. rhodesianum* sp. nov., *M. zatmanii* sp. nov., and *M. fujisawaense* sp. nov. Int J Syst Bacteriol 38:124–127 https://doi.org/10.1099/00207713-38-1-124.

12. Puri AW, Owen S, Chu F, Chavkin T, Beck DAC, Kalyuzhnaya MG, Lidstrom ME. 2014. Genetic tools for the industrially promising methanotroph *Methylomicrobium buryatense*. Appl Environ Microbiol 81:1775–1781 https://doi.org/10.1128/AEM.03795-14.

13. Schlechter RO, Jun H, Bernach M, Oso S, Boyd E, Muñoz-Lintz DA, Dobson RCJ, Remus DM, Remus-Emsermann MNP. 2018. Chromatic Bacteria – A Broad Host-Range Plasmid and Chromosomal Insertion Toolbox for Fluorescent Protein Expression in Bacteria. Front Microbiol 9 https://doi.org/10.3389/fmicb.2018.03052.
